# Circadian variation in MGMT promoter methylation and expression predicts sensitivity to temozolomide in glioblastoma

**DOI:** 10.1007/s11060-025-05242-3

**Published:** 2025-10-22

**Authors:** Maria F. Gonzalez-Aponte, Yitong Huang, William A. Leidig, Tatiana Simon, Omar H. Butt, Marc D. Ruben, Albert H. Kim, Joshua B. Rubin, Erik D. Herzog, Olivia J. Walch

**Affiliations:** 1 Division of Biology and Biomedical Sciences, Department of Biology, Washington University in St. Louis, Campus Box 1137, One Brookings Drive, St. Louis, MO 63130 USA; 2 Department of Mathematical Sciences, Smith College, Northampton, MA 01063 USA; 3 Department of Neurosurgery, Washington University School of Medicine, St. Louis, MO 63110 USA; 4 Division of Oncology, Department of Medicine, Washington University School of Medicine, St. Louis, MO 63110 USA; 5 Divisions of Pulmonary Medicine and Biomedical Informatics, Cincinnati Children’s Hospital, Cincinnati, OH 45229 USA; 6 The Brain Tumor Center, Siteman Cancer Center, Washington University School of Medicine, St. Louis, MO 63110 USA; 7 Department of Pediatrics, St. Louis Children’s Hospital, Washington University School of Medicine, St. Louis, MO 63110 USA; 8 Department of Neuroscience, Washington University School of Medicine, St. Louis, MO 63110 USA; 9 Arcascope Inc., 4075 Wilson Blvd, Floor 8, Arlington, VA 22203 USA; 10 Department of Neurology, University of Michigan, Ann Arbor, MI 48104 USA

**Keywords:** GBM, TMZ, *MGMT* expression and promoter methylation, Chronotherapy, Chronodiagnosis

## Abstract

**Purpose:**

Recent studies show that glioblastoma (GBM) is more sensitive to temozolomide (TMZ) in the morning. In cells, inhibiting O6-Methylguanine-DNA-Methyltransferase (*MGMT*) abolished time-dependent TMZ efficacy, suggesting that circadian regulation of this DNA repair enzyme underlies daily TMZ sensitivity. Here, we tested the hypotheses that *MGMT* promoter methylation and protein abundance vary with time-of-day in GBM, resulting in daily rhythms in TMZ efficacy.

**Methods:**

We assessed daily rhythms in *MGMT* promoter methylation in GBM in vitro and retrospectively analyzed *MGMT* methylation status in human GBM biopsies collected at different times of day. Next, we measured MGMT and BMAL1 protein abundances in GBM cells collected at four-hour intervals. To understand the therapeutic implications of circadian variations in MGMT, we incorporated its daily rhythms into an in vitro mathematical model capturing interactions between MGMT, TMZ, and GBM DNA.

**Results:**

We found daily rhythms in *MGMT* promoter methylation and protein levels in GBM in vitro, and in patient biopsies peaking at midday. Further, MGMT protein levels peaked at CT4, corresponding to the time of maximal TMZ efficacy in vitro. When we incorporated cell-intrinsic circadian rhythms in MGMT protein into a mathematical model for GBM chemotherapy, we found that dosing when daily MGMT levels peaked and began to decline produced maximum DNA damage.

**Conclusion:**

Our findings suggest that the likelihood of diagnosis of *MGMT* promoter methylation may vary with time of biopsy in GBM. Furthermore, theoretical modeling predicts that efforts to deliver TMZ after the daily peak of MGMT activity, with exact time being dose-dependent, may significantly enhance its therapeutic efficacy.

**Graphical Abstract:**

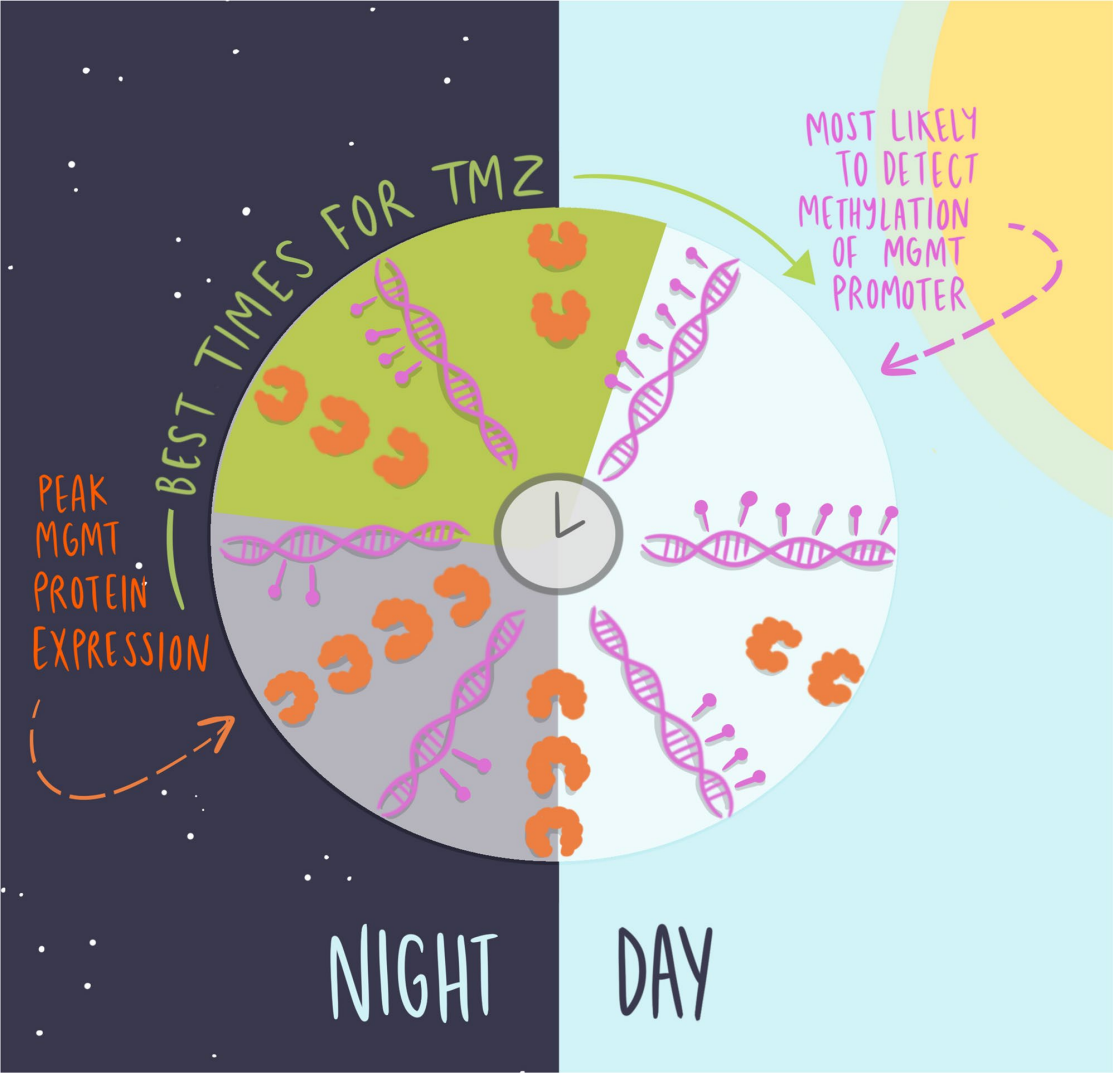

**Supplementary Information:**

The online version contains supplementary material available at 10.1007/s11060-025-05242-3.

## Introduction

Glioblastoma (GBM) is the most common primary malignant brain cancer with more than 300,000 adults diagnosed globally each year and a median survival of 15–18 months post-treatment [1, 2]. The current standard of care for GBM involves maximal safe surgical resection of the tumor, followed by concurrent radiation and chemotherapy with temozolomide (Temodar^Ⓡ^, TMZ), and tumor-treating fields [3, 4]. TMZ was introduced into the standard of care for GBM in 2005, and was found to induce the death of GBM cells by methylating O6-guanine, N7-guanine, and N3-adenine bases in DNA [5], leading to an extension of 2.5 months in patient overall survival [6, 7]. Unfortunately, response to TMZ is not universal, as the most frequent TMZ-induced cytotoxic lesion, O6-methylguanine (O6-MeG), can be repaired by the DNA repair enzyme O6-Methylguanine-DNA Methyltransferase (MGMT) in tumors that express this protein [5]. Thus, the presence or absence of *MGMT* is considered a prognostic factor for GBM patients, as tumor cells with epigenetic silencing of the *MGMT* promoter (i.e., *MGMT*-methylated) are more susceptible to DNA damage than those that express *MGMT* (i.e., *MGMT*-unmethylated) [6, 8, 9]. Based on this, prevailing clinical practice suggests that GBM patients with truly unmethylated *MGMT* promoter show limited benefit from TMZ treatment [10]. However, recent work has revealed that *MGMT* promoter methylation is not static. Rather, GBM cells have intrinsic daily fluctuations in *MGMT* promoter methylation and by extension, sensitivity to TMZ [11]. This led us to hypothesize that determining *MGMT* methylation status may be biased by biopsy time-of-day.

Rhythms in *MGMT* methylation and gene expression suggest that MGMT protein may vary over the course of the day and thus, treatment efficacy may be improved with dosing timed in accordance with MGMT activity [11–13]. Evidence for this can be found in a previous single-institute retrospective clinical study from our group, in which delivering TMZ in the morning (AM) increased overall survival by six months, compared to evening (PM) treatment, particularly in GBM patients with *MGMT*-methylated tumors [13]. Specifically, the effect sizes of AM vs. PM dosing in patients with *MGMT*-methylated tumors are comparable (> two months increase in overall survival) to the effect size of AM dosing vs. not taking TMZ at all, suggesting that dosing at the wrong time of day could have close to no benefit for these patients [13, 14]. These findings were recently recapitulated by our group in cellular and orthotopic models of GBM, where six timepoints for TMZ dosing were assessed and dosing with TMZ at the daily peak of circadian clock gene *BMAL1* expression was associated with significantly increased tumor cell death [11, 15]. Importantly, time-dependent sensitivity to TMZ was abolished when treating GBM tumors with an inhibitor of MGMT, suggesting that daily expression of *MGMT* modulates sensitivity to chemotherapy with TMZ [11]. A recent retrospective analysis also found time-of-day effects of TMZ on progression-free survival in *MGMT*-unmethylated GBM patients; however, that study differed in their definition of the morning group (i.e., midnight to 11am) and failed to find time-of-day effects in *MGMT*-methylated patients and on overall survival [16]. These results motivate us to better understand the underlying mechanisms of TMZ efficacy, and the potential role played by daily rhythms in *MGMT* expression and activity.

Here, we test the hypotheses that rhythms in *MGMT* promoter methylation and protein expression regulate time-dependent TMZ vulnerability and can influence methylation status diagnosis. We find the likelihood of detecting *MGMT* promoter methylation in human biopsies peaks around midday, suggesting methylation status diagnoses may be biased by the time of sample collection. Further, we find that MGMT protein abundance peaks in the early subjective morning in GBM cells in vitro, at Circadian time 4, near the minimum of *MGMT* gene expression and at the time when TMZ efficacy is higher, as found in our previous studies assessing daily TMZ efficacy at multiple timepoints [11]. To understand why maximum TMZ efficacy can occur when delivered in phase with maximum MGMT protein abundance, we incorporate the observed daily activity of MGMT into a simple in vitro mathematical model and find that TMZ-induced DNA damage can be maximized by dosing as MGMT levels are near their peak and beginning to decrease, with best times dependent on dose. Altogether, our theoretical model suggests that optimal TMZ-induced cell death may occur when dosing in the subjective early morning.

## Materials and methods

### Experimental methods

#### Glioblastoma cell culture

LN229 cells, a female human GBM cell line obtained from the American Type Culture Collection, were cultured as a monolayer in coated T-75 flasks (Nunclon Delta coated, Fisher) using DMEM/F12 Glutamax (Thermo Fisher) supplemented with 5% fetal bovine serum (FBS, Fisher) and 1% Pen/Strep (Thermo Fisher). Cells were maintained in a 5% CO2 incubator at 37 °C. Passage number in all experiments ranged from six to twelve.

Glioma 261 cells (GL261, obtained from the Division of Cancer Treatment and Diagnosis Tumor Repository of the National Cancer Institute), a male murine model of GBM, were cultured in monolayer in coated T-75 flasks (Nunclon Delta coated, Fisher) using RPMI-1640 (Sigma-Aldrich) supplemented with 10% FBS (Fisher), 1% L-Glutamine (Thermo Fisher), and 1% Pen/Strep (Thermo Fisher). Cells were maintained in a 5% CO2 incubator at 37 °C. Passage number in all experiments ranged from six to twelve.

Nf1^−/−^DNp53 male astrocytes (Generous Gift of Dr. Joshua Rubin, Washington University in St. Louis), a syngeneic murine model of GBM, were cultured in monolayer in coated T-75 flasks (Nunc Treated EasYFlasks, Fisher) using DMEM/F12 media (Gibco), supplemented with 10% FBS (Fisher) and 1% Pen/Strep (Thermo Fisher). Cells were grown in a 37 °C incubator with a 5% CO2 environment. Passage number in all experiments ranged from five to ten.

Primary human B165 (*MGMT*-methylated, male, Generous Gift of Dr. Albert Kim, Washington University in St. Louis) were cultured as spheres in 100 mm uncoated petri dishes (Fisher) using DMEM/F12 Glutamax media (Gibco), supplemented with 1% Pen/Strep (Thermo Fisher), 2% B27 (Miltenyi Biotec), 2.5 µg/mL heparin (Sigma-Aldrich), 20 ng/mL EGF (PeproTech), and 20 ng/mL bFGF (PeproTech). Cells were grown in a 37 °C incubator with a 5% CO2 environment. Passage number in all experiments ranged from six to ten.

#### BMAL1 knockdown

To knockdown *BMAL1* in LN229 cells, a predesigned shRNA plasmid packaged into a lentivirus (vector pLKO.1, viral titer = 10^6^ particles/mL) was obtained from Sigma Aldrich (target sequence: GCAGAATGTCATAGGCAAGTT). Cells were grown in T-25 cm^2^ flasks for 24 h and incubated for 10 min at 37 °C in 3 mL complete DMEM media with 10% FBS (Thermo Fisher), 5% Pen/Strep (Thermo Fisher), and 15 mg polybrene (Millipore #TR-1003-G). Following incubation, 5–10 mL of virus stock solution was added to each culture. Media was changed after 24 h and cells were kept at 37 °C, 5% CO2. After infection, cells were selected using puromycin (1.5 mg/mL, Thermo Fisher) for ten days. Knockdown efficiency was quantified by qPCR.

#### Quantitative real-time PCR (qRT-PCR) and quantitative methylation specific PCR (qMSP)

Genomic DNA (gDNA) was extracted and purified from 500,000 cultured LN229, LN229-*BMAL1* KD, B165, and Nf1^−/−^DNp53 GBM cells, synchronized by a media change [11, 17], and collected at CT4 or CT16 using the DNeasy Blood and Tissue Kit (Qiagen), according to the manufacturer’s instructions. The purified gDNA underwent bisulfite conversion using the EZ DNA Methylation-Gold Kit (Zymo Research), following the protocol provided by the manufacturer. The concentration and purity of gDNA was measured at each step using a NanoDrop spectrophotometer (Fisher Scientific). Gene expression changes were further probed using iTaqTM Universal SYBR Green Supermix (Bio-Rad). *MGMT*-unmethylated and Beta-actin primers, as well as non-converted DNA, were used as internal controls. Universal primers recognizing both methylated and unmethylated Beta-actin sequences were used. The following primer sequences were used: *MGMT*-methylated forward 5′-TTTCGACGTTCGTAGGTTTTCGC-3′, *MGMT*-methylated reverse 5′-GCACTCTTCCGAAAACGAAACG-3′, *MGMT*-unmethylated forward 5′-TTTGTGTTTTGATGTTTGTAGGTTTTTGT-3′, *MGMT*-unmethylated reverse 5′-AACTCCACACTCTTCCAAAAACAAAACA-3′, Beta-actin forward 5′-CTTCGCGGGCGACGAT-3′, and Beta-actin reverse 5′-CCACATAGGAATCCTTCTGACC-3′. qPCR amplification was carried out at 40 cycles with 10 ng of bisulfite treated gDNA in triplicates. Protocol is as follows: Cycle 1: 95 °C for three minutes; Cycle 2: 95 °C for 30 s Cycle 3: 60 °C for 30 s; repeat cycles 1–3 for 39 more times; Cycle 4: 72 °C for 1 min. Negative controls included no template DNA samples. All procedures were done in triplicate in three biological replicates.

#### Immunostaining and microscopy analysis

Cells were grown in 35 mm dishes containing a glass coverslip, synchronized by a media change [11, 17], and fixed every 4 h for 24 h, starting 24 h after initial plating, using 4% PFA for 10 min. Circadian time (CT) was defined relative to expression of the clock gene *Per2*, where CT0 corresponded to peak *Per2* expression [11, 17]. Cells were permeabilized for 30 min with 3% Triton-X (Millipore Sigma) in 1x PBS, and blocked for 1 h with solution containing 10% BSA (Sigma) and 0.3% Triton-X. Mouse anti-MGMT (1:500, Invitrogen, Waltham, MA) and rabbit anti-BMAL1 (1:500, Abcam, Cambridge, MA) were diluted in 2% blocking solution and incubated overnight at 4 °C. Samples were then rinsed three times with 1x PBS and incubated in secondary antibody solution (1:500 Alexa 488 goat anti-mouse IgG, and 1:500 Alexa 647 donkey anti-rabbit IgG, Abcam, Cambridge, MA) in 2% blocking solution for 1 h at room temperature. Samples were rinsed three times in PBS, stained with ProLong Gold mounting medium with DAPI (Life Technologies, Carlsbad, CA), and stored in darkness at 4 °C until imaging. To ensure specificity of the primary antibodies binding to the antigen and the secondary antibody binding to the primary, we included control samples with no primary or secondary antibodies, respectively. Each experiment was done in triplicate, in two biological replicates. Six imaging frames were collected and averaged per dish. Microscopy analysis was performed using ImageJ software. Quantification of protein expression was performed as corrected total cell fluorescence (CTCF) = Integrated Density – (Area of selected cell * Mean fluorescence of background readings), as described by McCloy et al., 2014.

#### In vitro cell growth assays and pharmacology with daily TMZ

GBM cells were plated at the same density (100,000 cells/well of a 6-well plate), synchronized by a media change [11, 17], and grown for 48 h to allow for attachment and growth. Cells were then treated with one of three TMZ concentrations (10, 100, 1000 µM) or vehicle (DMSO, 0.2%), at either Circadian time 4 (CT4) or CT16. Circadian time was defined relative to expression of the clock gene *Per2*, where CT0 corresponded to peak *Per2* expression [11, 17]. Cells were fixed after 72 h with 4% paraformaldehyde (PFA) and stained with 4′,6-diamidino-2-phenylindole (DAPI, 2 mg/mL). We chose to measure survival three days after TMZ administration to allow for approximate two-three cycles of cell division and TMZ-induced DNA lesions, as done previously [11]. DAPI fluorescence was quantified with the Infinite 200 PRO plate reader (V_3.37_07/12_Infinite, Tecan Lifesciences), and fluorescence was converted to cell number using a linear regression equation (Y = 0.009201*X + 2153) obtained from a standard curve where DAPI fluorescence was measured from different number of plated cells. We calculated the percent of cell death as 100 minus percent cell survival (percent cell survival = number of living cells treated with TMZ divided by the number of living cells treated with vehicle). All procedures were done in triplicate in three biological replicates.

#### Statistical analysis

The Jonckheere-Terpstra-Kendall (JTK) cycle algorithm, a non-parametric test used to distinguish between rhythmic and non-rhythmic data, was used to assess circadian rhythmicity in MGMT protein expression in vitro and the probability of *MGMT* promoter methylation in patient biopsies. We used *p* < 0.05 to designate significant circadian rhythmicity [18]. To avoid compromising the assumption of roughly even coverage, we limited our analysis to times when we had 6–15 biopsies. In addition, we applied the Rayleigh statistical test to evaluate differences in the number of patients with GBM scores of methylated or unmethylated as a function of time of day. Statistical significance of mean differences was determined by either Student’s t tests or two-way analysis of variance (two-way ANOVA) with multiple comparisons and Šídák’s post hoc test. All statistical analyses were performed in Prism (version 10.3.0).

### Retrospective analysis of MGMT promoter status in GBM biopsies

To determine if *MGMT* promoter methylation status varied by time of day in human GBM patients, we performed a retrospective chart review on patients diagnosed with GBM at Barnes-Jewish Hospital/Siteman Cancer Center. Inclusion criteria included all patients with newly diagnosed WHO Grade IV GBM (IDH-wildtype), diagnosed from January 2020 to October 2024 to ensure reliable determination of IDH status and *MGMT* promoter methylation status. A total of 302 patients met these inclusion criteria. Their clinical and demographic variables, such as *MGMT* promoter methylation status, age, sex, and time of surgery were collected. Time of surgery was determined by the recorded end time of anesthesia to approximate when the tissue samples were embedded for analysis. *MGMT* methylation status was determined by bisulfite PCR profiling of the *MGMT* promoter sequence. JTK cycle was used to assess circadian rhythmicity in *MGMT* promoter methylation status, with a level of *p* < 0.05 used to designate significant circadian rhythmicity [18].

### Modeling methods

To simulate the effects of TMZ on tumor DNA in vitro, the following system of differential equations was used:

$$\frac{{dD}}{{dt}} = {k_1}Z(1 - D) - {k_2}MD$$

$$\frac{{dM}}{{dt}} = - \alpha \sin (t\pi /12)$$

In this cell-based model, $\:Z$ represented TMZ toxin (MTIC) concentration (with dynamics taken from a previously published model), $\:M$ represented MGMT protein concentration, and $\:D$ represented the fraction of double-stranded damaged DNA in tumor cells in vitro [19]. We next chose to compare this model to a simpler model in which TMZ was modeled as an impulse followed by exponential decay (see Supplemental Figures S3-S6). Additionally, exploratory simulations were carried out using a modified equation for double-stranded DNA damage in which a Michaelis-Menten term was added (Supplemental Figures S7-S9), as well as model predictions using a non-sinusoidal daily waveform of MGMT production (Supplemental Figure S10-S12). Because findings did not significantly differ across models, the results from the cell-based model without the Michaelis-Menten term are presented in the main text and figures.

In this system of equations, $\:Z\:$created damaged DNA $\:D$ at rate $\:{k}_{1}$, in proportion to the amount of undamaged DNA, $\:1-D$. The parameter $\:{k}_{2}\:$removed damaged DNA in proportion to the amount of available MGMT and damaged DNA, $\:MD$. We adjusted the original model to include circadian variations in $\:M$ (i.e., MGMT protein) as a sinusoid with a period of 24 h, with initial conditions chosen such that it reached its maximum value 4 h after the start of the simulation, circadian time 4 (CT4), in keeping with previous experimental data. The MGMT derivative scalar $\:\alpha\:$ and initial condition $\:{M}_{0}\:$were chosen to enforce the timing of the maximum MGMT and a 10-fold change in $\:M$ over the course of the day. Finally, we assumed that the initial conditions for $\:Z$ and $\:D$ were $\:{Z}_{0}=0$ and $\:{D}_{0}=0$, respectively. This reduced our unknown parameters at baseline to $\:{k}_{1}\:$and $\:{k}_{2}$, corresponding to the relative strengths of TMZ at inducing DNA damage and MGMT at repairing it.

#### Cell death

To model TMZ-induced apoptosis, we assumed that the likelihood of cell death was proportional to the total DNA damage over time; $\:\int\:D\left(t\right)dt=\varvec{D}$. We also assumed that DNA damage exceeding threshold $\:{D}_{T}$ killed all cells. This fatal threshold $\:{D}_{T}\:$can be solved for uniquely using data that shows that 1000 µM TMZ demonstrates approximately 60% as much variation in dosing efficacy over the course of the 24 h day as 100 µM TMZ [11].

This in vitro model made the following assumptions: (1) The only factor affecting the impact of TMZ is the presence or absence of MGMT, (2) the apoptosis decision is based on cumulative DNA damage (the area under the TMZ-induced damage curve), and (3) the extent of variation of MGMT protein over the course of the day approximately agrees with the variation in the *MGMT* gene (10-fold difference between maximum and minimum).

To handle the unknowns around the parameters reflecting the relative strengths of TMZ vs. MGMT, we simulated a wide range of potential values. To find optimal dosing times for TMZ, the quality of the model fit was determined using the mean and variation (maximum - minimum) of cell death induced by TMZ across all times of day from published data [11]. By excluding information linking timing to TMZ efficacy in fitting this model, we aimed to predict time-of-day variations in TMZ efficacy based solely on first principles and the MGMT protein data reported here.

## Results

### GBM has daily rhythms in MGMT protein expression and promoter methylation

**Fig. 1:**
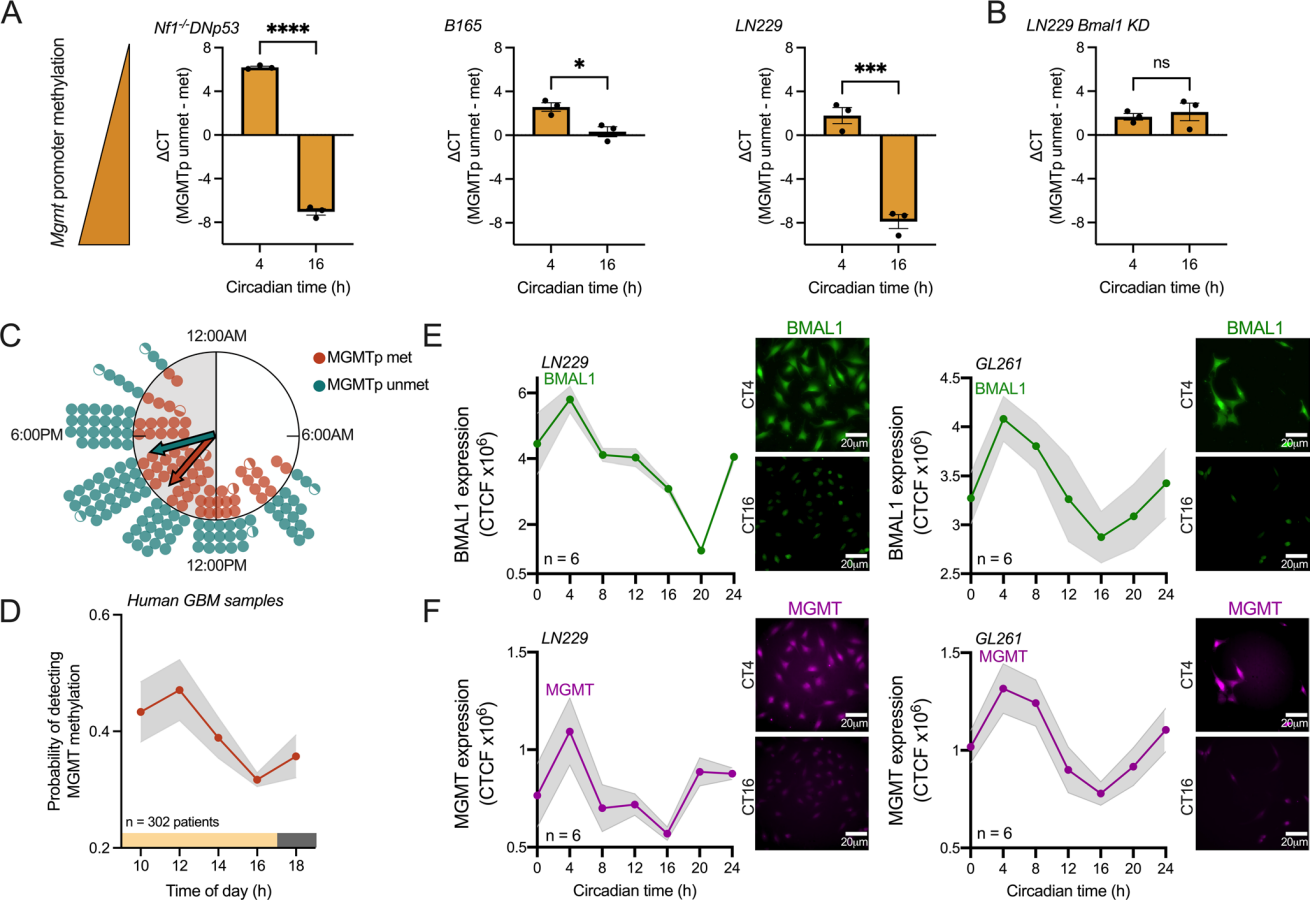
GBM exhibits daily rhythms in *MGMT* promoter methylation and protein abundance. (**A**) Murine Nf1^−/−^ DNp53, primary B165, and human LN229 GBM cells show daily rhythms in *MGMT* promoter methylation in vitro, with lower levels observed at circadian time 4 (CT4), and higher methylation observed at CT16 (*n* = 3 samples per time point, mean ± SEM, student’s t test, *p < 0.05, ***p < 0.001, ****p < 0.0001). (**B**) Human LN229 cells lacking a functional circadian clock (LN229 *BMAL1* KD) show no daily rhythms in *MGMT* promoter methylation in vitro, with both CT4 and CT16 time points showing low methylation levels (*n* = 3 samples per time point, mean ± SEM, student’s t test, ns *p* > 0.05). (**C**) Distribution of patients with GBM tumors scored as *MGMT* promoter methylated (MGMTp met, orange circles, peak time 3:00pm, arrow length = 0.839, Rayleigh test, *p* < 0.05) or unmethylated (MGMTp unmet, blue circles, peak time 5:00pm, arrow length = 0.842, Rayleigh test, *p* < 0.05) as a function of time-of-day of biopsy. In these Rayleigh plots, each filled circle equals data from two patients and half-circle from one patient, each arrow points to the mean time of day of the samples and the length of the arrow indicates the variation from the mean phase (0 = random, 1 = all patients methylated or unmethylated at the same time of day). (**D**) The probability of a tumor biopsy being identified as MGMTp methylated is higher around noon, and reaches a minimum (within the window tested) around 4:00pm (*n* = 302 patients, annual mean ± SEM over five years of tumor collection, average trace scored circadian by JTK cycle, *p* < 0.05). **E**-**F**) LN229 (left) and GL261 (right) cells show daily rhythms in BMAL1 (E, green) and MGMT (F, purple) protein expression, peaking at CT4 in both cell lines. Representative images of staining for BMAL1 and MGMT at CT4 (peak) and CT16 (trough) are shown (*n* = 6 samples per time point, mean ± SEM, scale bar = 20 μm, average trace scored circadian by JTK cycle, *p* < 0.05)

A recent study found that LN229 GBM cells have intrinsic circadian rhythms in sensitivity to TMZ and in *MGMT* promoter methylation, which controls the expression of the DNA repair enzyme MGMT [11]. Yet it is unknown whether other GBM models also show daily rhythms in *MGMT* promoter methylation. To test the hypothesis that *MGMT* promoter status varies with time of day in GBM cells of different backgrounds, we first assessed whether *MGMT* promoter methylation varies with circadian time in murine (Nf1^-/-^DNp53), human (LN229), and primary (B165) GBM cells in vitro. Consistent with previous studies [11] and across all GBM cell lines, we found lower *MGMT* promoter methylation when cells were collected in the subjective morning, at Circadian time 4 (CT4), and higher in the subjective evening, at CT16 (Fig. 1A). CT was previously defined relative to expression of the clock gene *Per2*, where CT4 and CT16 correspond to 4 and 16 h after peak *Per2* expression, respectively [11, 17]. To test if daily rhythms in *MGMT* promoter methylation depend on an intact circadian clock, we measured *MGMT* promoter methylation in LN229 cells with The Core circadian clock gene *BMAL1* knocked down (LN229 *BMAL1* KD). These cells have been previously shown to have disrupted circadian rhythms [15, 17]. We found that LN229 *BMAL1* KD cells show no daily rhythms in *MGMT* promoter methylation, with methylation levels being low at both circadian times (Fig. 1B), suggesting that the circadian clock regulates daily methylation of the *MGMT* promoter.

To determine if daily rhythms in *MGMT* promoter methylation impact diagnosis across the heterogeneity of GBM tumors, we next tested whether detecting *MGMT* promoter methylation changes with time of collection in patient biopsies. We evaluated *MGMT* promoter status from patient biopsies collected at different times of day, from 10am to 6pm, binned every two hours. We analyzed *MGMT* promoter status annually from a total of 302 GBM patients who underwent surgery between 2020 and 2024 (i.e., five patient cohorts separated by year of biopsy). We found the number of samples scored as *MGMT*-methylated peaked in biopsies collected around midday (Fig. 1C). We further found that the probability of detecting *MGMT* promoter methylation among all patient GBM samples showed a daily rhythm that peaked around noon (Fig. 1D, average trace scored circadian by JTK cycle, *p* < 0.05), varying by at least 20% over the course of the day.

Finally, we evaluated whether GBM cells have daily rhythms in MGMT protein abundance. We used the well-characterized human LN229 and murine GL261 GBM cells, which have been previously found to have reliable circadian rhythms in clock gene expression and response to TMZ chemotherapy [11, 17]. We fixed GBM cells every 4 h over 24 h and performed immunostaining for MGMT and BMAL1 proteins (Figures S1 and S2). In both human and murine GBM cell lines, we found daily rhythms in expression of the clock protein BMAL1 (Fig. 1E) and MGMT (Fig. 1F) peaking during the subjective morning, at CT4 (average traces scored circadian by JTK cycle, *p* < 0.05). The amplitude and phase of these protein rhythms are consistent with a previous report of *MGMT* and *BMAL1* mRNA and promoter methylation rhythms in GBM cells in vitro [11]. We conclude that *MGMT* methylation status and MGMT abundance in GBM appear to vary with time of day of sample collection.

### Mathematical modeling predicts maximal TMZ efficacy in the early subjective morning

To further evaluate the potential mechanisms underlying our counterintuitive finding that MGMT protein abundance reaches its daily maximum close to the time of maximal TMZ sensitivity [11], we incorporated the daily rhythms in *MGMT* promoter methylation and protein abundance into a published mathematical model [19]. This cell-based model uses differential equations to describe the dynamics of in vitro DNA damage and predict the time of day when TMZ efficacy would be maximized. The model includes conversion of TMZ to its toxic form, MTIC, in the nucleus. We adapted this model to incorporate daily rhythms in MGMT, where dynamics are driven by$\:\:{k}_{1}$, which represents the strength of TMZ (i.e., how effectively it damages DNA), and $\:{k}_{2}$, which represents the strength of MGMT (i.e., how effectively it repairs DNA).

**Fig. 2:**
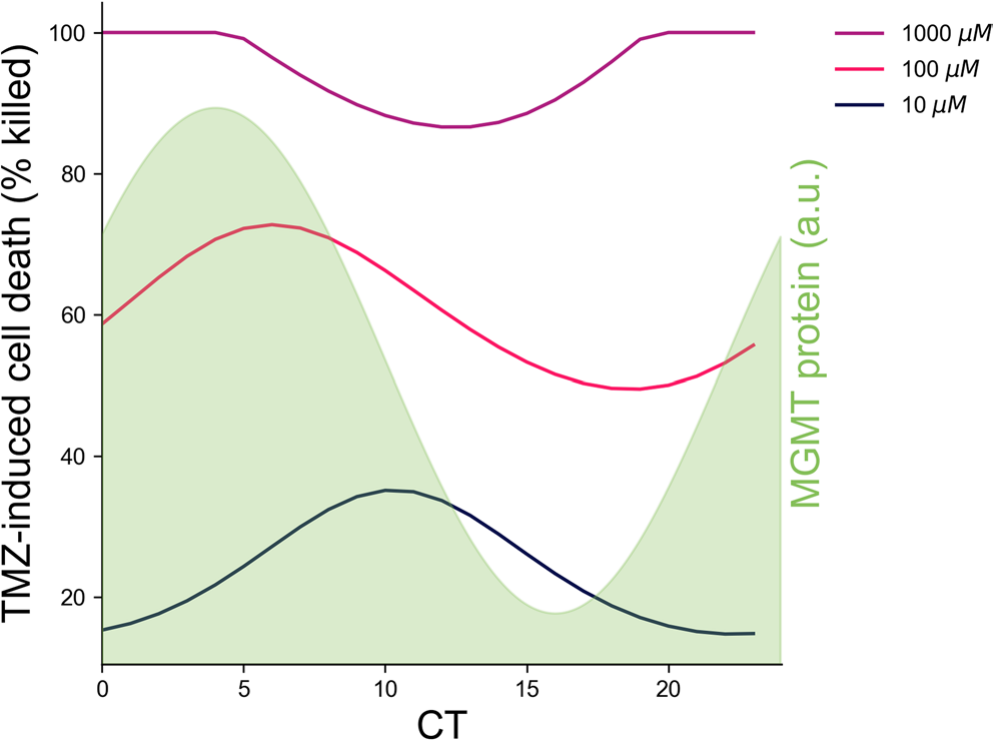
Theoretical efficacy of TMZ-induced cell death depends on dose and time after the daily peak of the MGMT protein. A cell-based mathematical model predicted that the percent of GBM cell death in vitro varies following TMZ delivery at different circadian times (CT). Efficacy of 100 µM TMZ (pink line) showed a higher amplitude daily rhythm compared to 10 µM (black line) and 1000 µM (purple line) TMZ, with a peak closely after the maximum in MGMT protein abundance (green shaded background), with the timing of the peak MGMT chosen to agree with the data in Fig. 1F. In this theoretical model, TMZ induced around 73% cell death when delivered shortly following maximum MGMT abundance (CT6), while delivering when MGMT levels began to rise (CT16) induced around 49% cell death

To avoid biasing model fits towards a best or worst time for TMZ administration, we ignored timing information from our previous six-timepoint experiments in [11], and instead fit only the mean and range of variation in cell death for each dose. Using values for $\:{k}_{1}$, and $\:{k}_{2}\:$ which best fit the observed mean and range of variation of the cell death data in [14] $\:{(k}_{1}=12.54,\:{k}_{2}=0.818,\:and\:{D}_{T}=344)$, the model predicted that 73% cell death would occur with TMZ treatment around CT4-6 (i.e., subjective early morning), when MGMT abundance begins to decline, while 49% cell death would occur when treated at CT16-20 (i.e., subjective evening), as MGMT abundance begins to rise (Fig. 2), in a dose-dependent manner. Nearly identical results were seen for a simpler model (Fig. S4), while the same best time with even greater time-of-day variation was observed when a Michaelis-Menten term was added to the in vitro cell model (Figure S7).

As they were designed to, all models were able to demonstrate the phenomenon observed in our prior work that 10 µM and 1000 µM TMZ both have smaller time-of-day variations in efficacy than 100 µM, with 10 µM TMZ having reduced time-of-day variation due to the fact that, as a smaller dose, it causes less DNA damage than 100 µM at all times, while 1000 µM TMZ reaches a threshold where *all* cells are killed, regardless of time of day (Figs. 2, S4, S7).

**Fig. 3:**
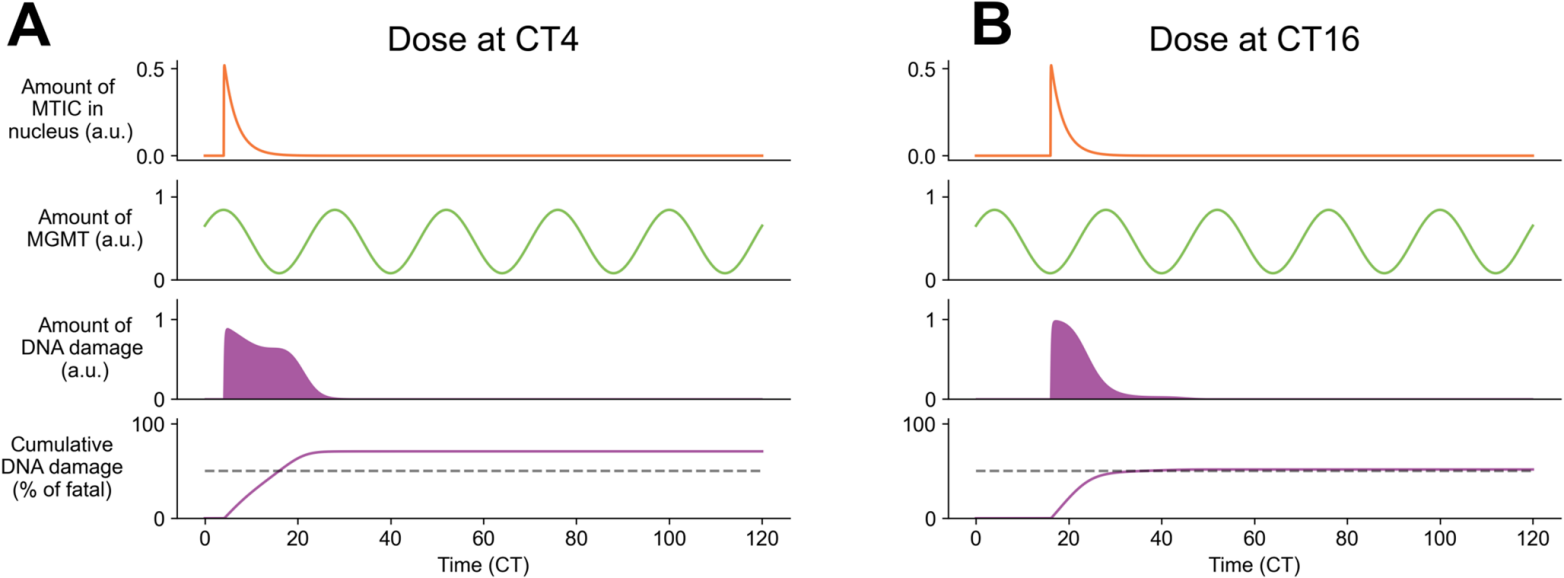
A theoretical model highlights changes in TMZ-induced DNA damage and cell death as a function of the daily rhythm in MGMT protein abundance. (**A**) In vitro mathematical modeling that incorporates daily rhythms in TMZ delivery (first row) and MGMT protein abundance (second row) predicted higher DNA damage (third row) and cumulative cell death (fourth row) when TMZ was delivered at CT4. The model predicted that TMZ at CT4, but not CT16, induced cumulative DNA damage that exceeded a 50% threshold (dashed line), leading to maximum TMZ-induced GBM cell death. (**B**) Dosing at CT16, which corresponds to lowest MGMT protein abundance, yielded less DNA damage and cell death in the model. The amount of predicted DNA damage lasted for a shorter time and barely crossed a 50% threshold, leading to reduced prediction of TMZ-induced GBM cell death. The top row in each plot shows the time profile of the toxic form of TMZ (MTIC) in the nucleus, the second row shows daily MGMT protein, the third shows the damage to the DNA, and the fourth shows cumulative damage to the DNA, scaled by the fatal threshold $\:{D}_{T}$. Parameters were $\:{k}_{1}=12.54$, $\:{k}_{2}=0.818$, and $\:{D}_{T}=344$

Using the same parameters that best fit the data in [11], we found that the time of TMZ arrival at the nucleus influenced the time course of DNA damage, with more cumulative DNA damage occurring when TMZ was delivered near or shortly after the daily maximum of MGMT and BMAL1 protein expression (CT4, Fig. 3A), compared to when MGMT protein was at its daily minimum (CT16, Fig. 3B). Together, these theoretical results suggest that an intermediate dose of TMZ timed according to daily rhythms in MGMT abundance in GBM may result in greater cell death and maximum chemotherapy efficacy in vitro.

**Fig. 4:**
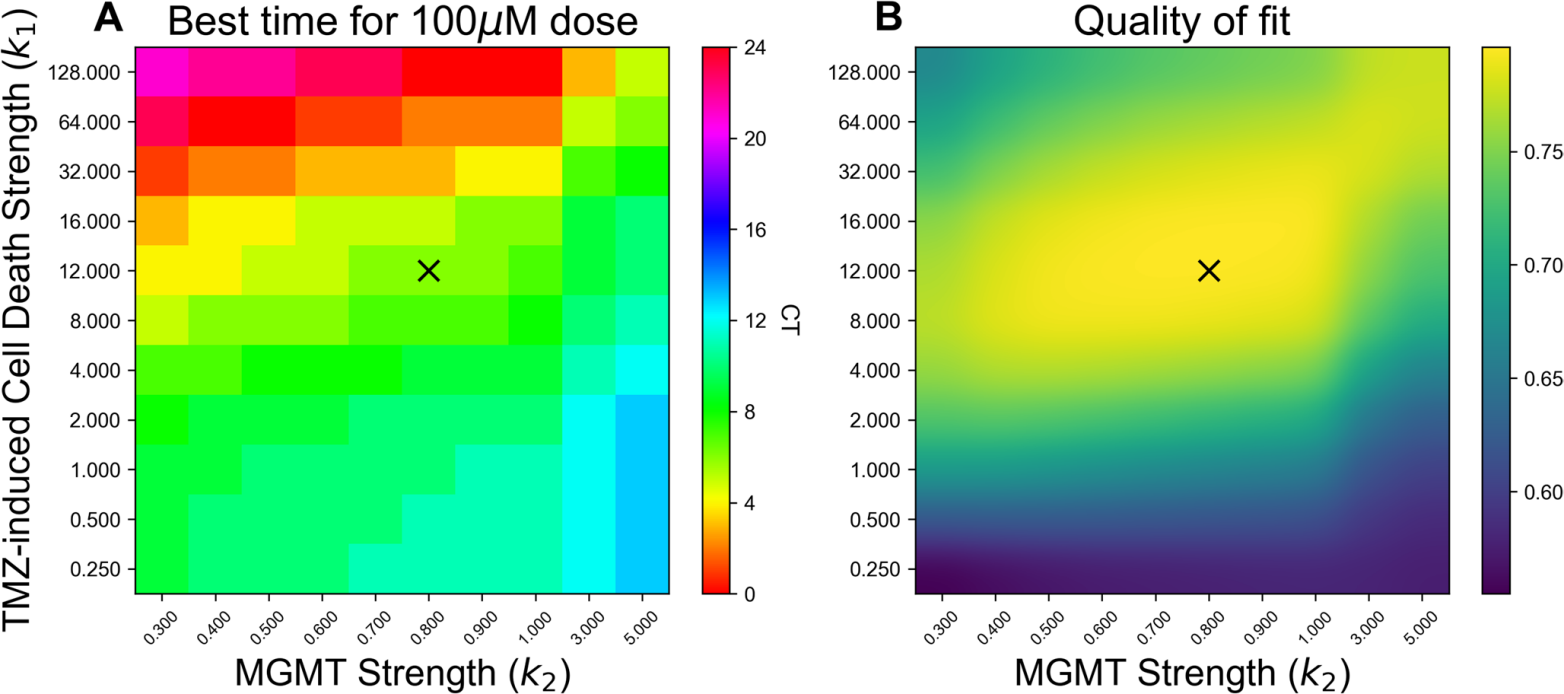
A theoretical model shows early morning treatment with TMZ maximized efficacy and cell death, regardless of TMZ and MGMT strength. (**A**) Modeling the in vitro strength of TMZ-induced DNA damage (y-axis) relative to the daily ability of MGMT to repair DNA (x-axis) showed that, across all the parameter sets tested that corresponded to high quality fits (yellow regions in B) to the mean and range of cell death observed in previous experimental data, the dose time that maximized cell damage was near the peak of MGMT (CT4) or on its descent, corresponding to subjective morning dosing. No optimal TMZ dosing times were found near the lowest levels of MGMT abundance (dark blue, purple in color bar). (**B**) Heatmap showing which parameter choices best matched the mean DNA damage for different doses and the range of cell death variation in previously reported data. Yellow indicates a closer fit to the data, while blue indicates a poor fit. Across all the parameter sets tested, optimal TMZ dosing was predicted to be in a subjective morning treatment from our theoretical model. The X marks in A and B correspond to the parameter set that best matches the mean cell death and range of cell death observed in experimental data following 100 µM TMZ treatment, with best fit at CT6

The surprising finding of earlier-than-expected optimal dosing led us to further test the robustness of the optimal time for TMZ treatment with different choices of TMZ and MGMT rate constants, $\:{k}_{1}\:$and $\:{k}_{2}$, (solving for $\:{D}_{T}$ to match previously data from [14] for each $\:{k}_{1}$, $\:{k}_{2}$ pair). Across all parameter pairs assessed, we found that the best time to dose with TMZ ranged from CT0 to CT12, with no optimal times occurring near MGMT protein’s lowest expression at CT16 (Fig. 4A), despite the fact that dosing near MGMT protein’s minimum seems intuitively like an ideal time to dose. The parameters which best fit the data (Fig. 4B, higher values in yellow correspond to better fit), corresponded to maximum effect from TMZ dosing between CT2 and CT8. This is in agreement with our prior finding that maximum TMZ-induced tumor cell death occurs at CT4 [11], which corresponds to the time in the data presented here (Fig. 1F) when daily MGMT protein levels are near their peak. Results from a second model with fewer assumptions yielded similar results (Figures S3-S6), as did a version of the model with a Michaelis-Menten term added (Figures S7-S9), suggesting that 100 µM TMZ dosing as daily MGMT protein abundance begins to decrease may provide maximal efficacy against GBM growth.

In summary, our model predicted that TMZ efficacy on GBM cells in vitro may peak as MGMT protein is beginning to decrease. By extrapolation, based on the time of peak BMAL1 expression in mouse xenograft models of GBM [11, 17], the model suggests TMZ should be given around CT4, corresponding to treatment in the subjective early morning. When delivered in the subjective morning as MGMT abundance declined from its peak, a 100 µM dose of TMZ induced an additional 25–35% GBM cell death compared to delivering in the subjective afternoon/evening when MGMT abundance was low and beginning to increase (Figs. 2, S4, S7).

### GBM cells are more sensitive to TMZ when delivered at earlier circadian times

**Fig. 5:**
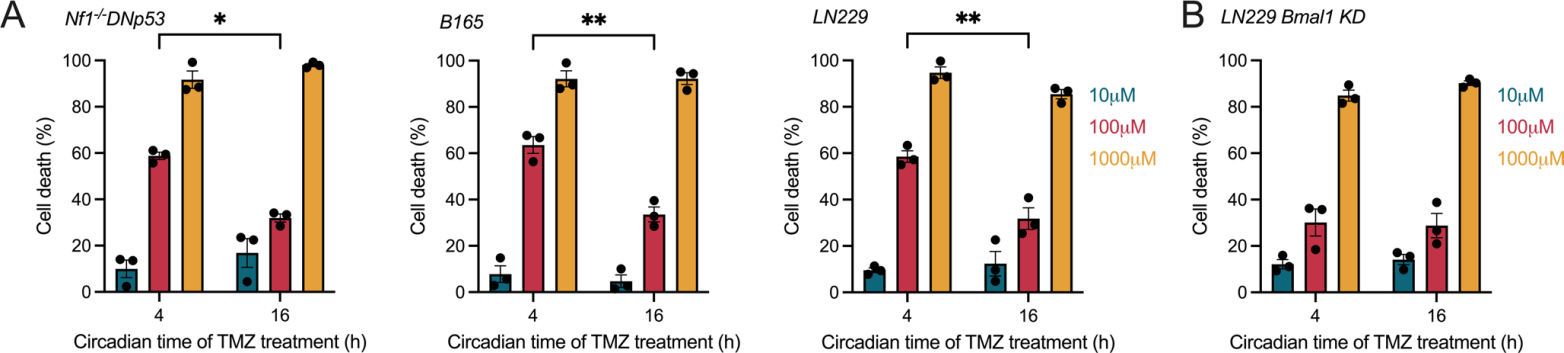
GBM TMZ sensitivity depends on circadian time of treatment in vitro. (**A**) Murine Nf1^−/−^ DNp53, primary B165, and human LN229 GBM showed higher cell death when treated with 100 µM TMZ (red bars) at CT4 compared to CT16. No daily rhythm was observed when treating cells with 10 (blue bars) or 1000 (yellow bars) µM TMZ (*n* = 3 per group, mean ± SEM, two-way ANOVA with Šídák’s multiple comparisons test, *p < 0.05, **p < 0.01). (**B**) LN229 cells lacking a functional circadian clock (LN229 *Bmal1* KD) had dose-, but not time-of-day, dependent responses to TMZ treatment (*n* = 3 per group, mean ± SEM, two-way ANOVA with Šídák’s multiple comparisons test, ns *p* > 0.05)

To test the predictions of our in vitro mathematical models, we treated murine (Nf1^-/-^DNp53), human (LN229), and primary (B165) GBM cell lines with three different doses of TMZ, previously tested for circadian effects [11], at either a model-predicted optimal (CT4) or suboptimal (CT16) circadian time. We found that TMZ dose-dependently induced cell death in a manner dependent on circadian time, consistent with the mathematical models. Specifically, we found that 100 µM TMZ induced an average of 60% cell death when delivered at CT4, compared to 30% at CT16, in all GBM cells in vitro (Fig. 5A). The daily rhythm in TMZ sensitivity was not detected at 10 or 1000 µM TMZ, with 10 µM inducing less than 10% and 1000 µM inducing 90% cell death. We next assessed if daily rhythms in sensitivity to TMZ depend on an intact circadian clock by treating LN229 *BMAL1* KD cells with TMZ at either CT4 or CT16. We found that knocking down *BMAL1* abolished the daily rhythm in TMZ sensitivity, with all doses yielding similar percentages of cell death regardless of time of day of treatment (Fig. 5B). We conclude that GBM sensitivity to TMZ in vitro increases around CT4, corresponding to when daily MGMT and BMAL1 protein abundance begins to decline.

## Discussion

Our results suggest that, across multiple GBM cell lines, *MGMT* promoter methylation levels change with time of day of collection, with lower methylation levels observed in the subjective afternoon in vitro. Consistent with this, we find that the probability of detecting *MGMT* promoter methylation significantly decreases from around 50% to 30% when patients were biopsied later in the day compared to in the morning. This timing and amplitude aligns with the reported daily oscillations in *MGMT* mRNA and promoter methylation in human GBM cells in vitro [11]. Taking into consideration the limitations of clinical scoring of *MGMT* methylation [20] and this retrospective analysis, future clinical trials should be designed to better control for variables like the time of sample fixation, tumor processing times, method for determining *MGMT* status [21], tumor location, anesthesia duration, as well as other comorbidities like patient age and sex. Because this analysis was constrained by the typical hours of the day when biopsies occur, we do not know what happens to *MGMT* promoter methylation from around 6pm until 10am. Further, to account for interpersonal differences in circadian rhythms (i.e., chronotype), it will be important to measure how *MGMT* promoter methylation and expression varies with time of day in individual GBM patients. Finally, *MGMT* methylation may be heterogeneous in a patient so that it will be important to test if there is nucleotide-specific circadian methylation of the *MGMT* gene in repeated biopsies and across cell types within a biopsy [22].

Daily rhythms in MGMT protein expression are likely driven by the circadian clock transcription factor, BMAL1, across the variety of GBM cells. We previously reported that *MGMT* mRNA levels peak around 12 h after peak *BMAL1* mRNA [11]. We now find that maximal BMAL1 protein aligns with the time of maximal transcription and translation of MGMT. We posit that BMAL1 promotes *MGMT* expression, which is rapidly translated into MGMT protein. Altogether, these results suggest that cell-intrinsic circadian rhythms regulate MGMT abundance and promoter methylation so that scoring *MGMT* status on GBM biopsies varies with time of sample collection. Considering that conclusions about promoter methylation based on four GBM cell lines may not fully capture the heterogeneity of primary and recurrent GBM including in their circadian gene expression [22–24] and the clinical challenges posed by repeating biopsies at multiple times of day, approaches like focused ultrasound-enhanced liquid biopsies, ex vivo organotypic patient-derived slice cultures, or patient-derived tumor spheroids could accurately and less-invasively measure daily *MGMT* expression and promoter methylation in individual patient samples [21, 25, 26].

Our experimental data suggests, surprisingly, that the best time to dose with TMZ aligns with the daily peak of MGMT protein abundance even though MGMT opposes chemotherapy by repairing TMZ-induced DNA damage. To understand this counterintuitive result, we built a simple mathematical model of TMZ dynamics by extending a previously published in vitro model. Our model found that, across all parameters tested, it was never optimal to dose at the minimum of MGMT abundance. The parameter values which best captured the mean cell death and range of cell deaths observed in previous experimental data yielded CT2 - CT8 as the most effective times to dose with 100 µM TMZ [11]. Because TMZ must be converted to its toxic form and subsequently alkylate and damage DNA, it takes many hours from TMZ administration to peak DNA damage, even in vitro. Delivery of TMZ when daily MGMT levels begin to decline is thus predicted to result in more DNA damage left unrepaired and greater likelihood of cell death.

Our model structure reproduces the result that 100 µM TMZ has higher amplitude daily efficacy rhythms relative to 10 µM and 1000 µM doses from previous results [11]—or “windows of circadian effect”—where low doses show minimal circadian variation because of the noise floor while high doses show minimal circadian variation because of ceiling effects. The model is also able to reproduce the new finding from this paper that the 100 µM dose is most effective as MGMT levels fall from their daily peak. We find that this result holds true for all tested parameter values, which include TMZ strength ($\:{k}_{1}$), MGMT strength ($\:{k}_{2}$), and threshold of DNA damage at which cell death occurs ($\:{D}_{T}$). Lastly, we find that best time to dose depends on the amount of the drug being delivered, with the best times differing between 10 µM and 100 µM across all model variants tested.

The simple model allowed us to identify a minimal set of assumptions needed for in vitro TMZ efficacy to peak after the daily maximum of MGMT protein abundance. Further evaluation of these assumptions about TMZ pharmacokinetics and pharmacodynamics, MGMT-independent DNA repair, and details of how DNA damage induces apoptosis could improve the model. Future studies should also consider interactions between TMZ timing, survival, and side effects, and whether daily sensitivity to TMZ is changed under chemoradiation paradigms to better model the standard of care and potential translation of these findings into the clinic.

Our previous studies found that maximum *MGMT* methylation occurs at CT16 in GBM cells [11]. We now find that *MGMT* promoter methylation peaks 12 h after peak MGMT protein. Given that *MGMT* promoter methylation appears to peak around noon in human biopsies, we estimate that MGMT protein will peak in the middle of the night. This theoretical modeling suggests that dosing near the peak of MGMT and as it begins its decrease may be a better strategy compared to dosing near its trough. This suggests that the benefit to morning dosing seen in retrospective human data may have been driven primarily by patients who dosed in the early morning.

One limitation of this study is that we do not know the intratumoral concentration of TMZ when delivered at different circadian times. Previous studies have modeled TMZ pharmacokinetics and found that peak concentrations ranged from 14 to 35 µM to in human GBM, which best match clinically relevant doses (i.e., 75–200 mg/m^2^/day) [27]. In simulations and experiments, we found high amplitude daily rhythms in sensitivity to 100 µM TMZ. Simulation data predicted a lower amplitude rhythm in DNA damage to 10 µM TMZ and no rhythm to 1mM TMZ, suggesting that clinically relevant doses should be timed to the morning to obtain maximum chemotherapy efficacy. Future studies should measure the intratumoral concentration of TMZ when delivered at different circadian times and in combination radiation to further determine the optimal times to dose in the clinic.

Altogether, previous experimental data [11], the protein abundance data presented here, and our theoretical model suggest that dosing near the minimum of MGMT protein abundance means dosing right when repair mechanisms are gearing up again, blunting the effect of TMZ, while dosing near the maximum of MGMT or on the way down is equivalent to dosing at the start of a window of vulnerability when MGMT is lowered for a prolonged period and repair mechanisms are more quiescent. Our findings have significant implications for both chronodiagnosis and chronomodulation of therapies for GBM. While *MGMT* methylation may be thought of as a binary measurement with confounds, where methylation leads to gene silencing and unmethylation leads to increased *MGMT* expression, our results suggest that this process may be more dynamic and, in part, regulated by the circadian clock. If there exists, for instance, a daily *MGMT* methylation rhythm across individuals, with some having low or high methylation levels, it may be that the only individuals being diagnosed as *MGMT* methylated are those with sufficiently high amplitude expression levels and who are biopsied near the *MGMT* methylation peak. Clinically, this may suggest that many individuals may be mischaracterized as having an unmethylated or methylated tumor because of the time of day when samples were collected, which could in turn affect prognosis and optimal treatment strategies to deliver chemotherapy with TMZ.

Recent studies argue that patients with *MGMT*-unmethylated GBM tumors show limited benefit of TMZ but have not considered circadian time as an important variable for both measuring *MGMT* methylation and assessing TMZ efficacy [10]. Previous studies have found that delivering TMZ in the morning yields a six-month increase in overall survival, specifically for patients diagnosed with *MGMT*-methylated GBM tumors, with no changes in drug safety or side effects [13, 14]. However, we do not know whether those patients exhibited a daily rhythm in *MGMT* methylation. We found that *MGMT* methylation diagnoses in humans varied from 50% when biopsied early in the day to 30% when collected later in the day. This has both significant clinical and therapeutic implications. This diagnosis contributes to the prognosis and therapeutic approach a patient will receive. Treating *MGMT*-unmethylated tumors remains an elusive goal. If *MGMT* methylation is circadian in the heterogeneous cells of GBM, it becomes a therapeutic target to deliver TMZ at times when it is unmethylated and, perhaps, to lock the cells at a circadian phase when methylation is physiologically low.

## Supplementary Information

Below is the link to the electronic supplementary material.

Supplementary Material 1

## Data Availability

All data and code reported in this paper will be shared upon request by the corresponding authors.
