# Supplementary material for: Circadian variation in MGMT promoter methylation and expression predicts sensitivity to temozolomide in glioblastoma
Source: J Neurooncol. 2025 Oct 22;176(1):36. doi: 10.1007/s11060-025-05242-3 (PMC12546296; doi:10.1007/s11060-025-05242-3)
Supplement: Supplementary file 1 — Supplementary Material 1 [file 11060_2025_5242_MOESM1_ESM.pdf]

Title: Circadian variation in MGMT promoter methylation and expression predicts sensitivity to temozolomide in glioblastoma

Authors: Maria F. Gonzalez-Aponte[1], Yitong Huang[2], William A. Leidig[3], Tatiana Simon[1], Omar H. Butt[4], Marc D. Ruben[5], Albert H. Kim[3,6], Joshua B. Rubin[7,8], Erik D. Herzog[1*], Olivia J. Walch[9,10*]

Affiliations:

[1] Department of Biology, Division of Biology and Biomedical Sciences, Washington University in St. Louis, St. Louis, MO, 63130, USA

[2] Department of Mathematical Sciences, Smith College, Northampton, MA, 01063, USA

[3] Department of Neurosurgery, Washington University School of Medicine, St. Louis, MO, 63110, USA

[4] Division of Oncology, Department of Medicine, Washington University School of Medicine, St. Louis, MO, 63110, USA

[5] Divisions of Pulmonary Medicine and Biomedical Informatics, Cincinnati Children's Hospital, Cincinnati, OH, 45229, USA

[6] The Brain Tumor Center, Siteman Cancer Center, Washington University School of Medicine, St. Louis, MO, 63110, USA

[7] Department of Pediatrics, St. Louis Children's Hospital, Washington University School of Medicine, St. Louis, MO, 63110, USA

[8] Department of Neuroscience, Washington University School of Medicine, St. Louis, MO, 63110, USA

[9] Arcascope Inc., Arlington, VA, 22203, USA

[10] Department of Neurology, University of Michigan, Ann Arbor, MI, 48104, USA

***Co-Corresponding authors:**

- **Name:** Erik D. Herzog
- **Mailing address:** Department of Biology, Washington University in St. Louis, Campus Box 1137, One Brookings Drive, St. Louis, MO, 63130, USA
- **Phone: (**314) 935-8635
- **E-mail address:** herzog@wustl.edu

- **Name:** Olivia J. Walch
- **Mailing address:** Arcascope Inc., 4075 Wilson Blvd, Floor 8, Arlington, VA, 22203, USA
- **Phone:** (252) 922-0397
- **E-mail address:** olivia@arcascope.com

ORCID:

MFGA: 0000-0002-8139-312X

YH: 0000-0002-5200-8077

WL: 0009-0008-3127-3905

TS: 0000-0001-5686-8798

OHB: 0000-0003-2252-3673

MDR: 0000-0002-7893-0238

AHK: 0000-0002-1751-8493

JBR: 0000-0002-7395-1937

EDH: 0000-0001-7209-5174

OJW: 0000-0002-7881-6910

Supplementary Materials

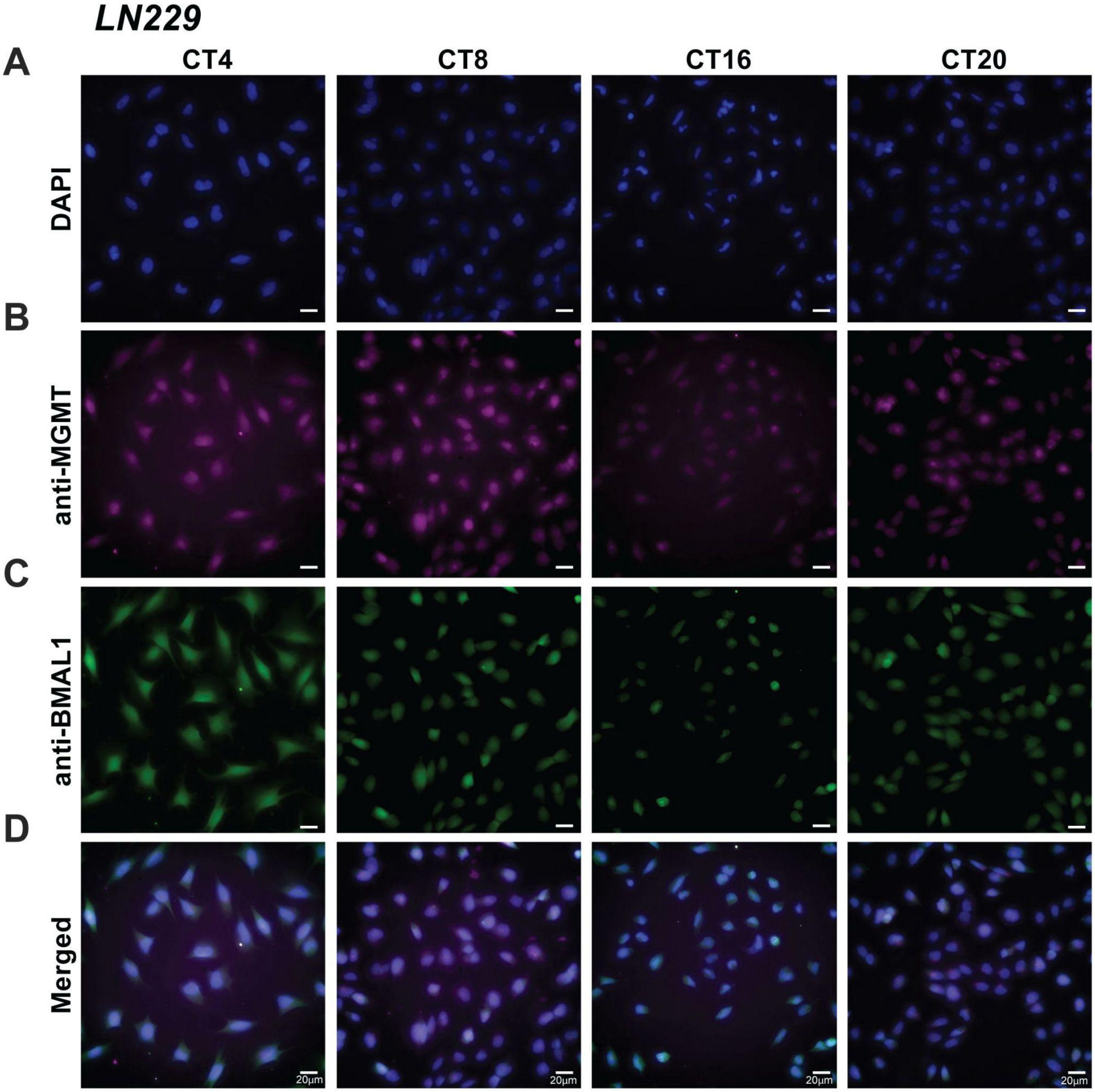

Supplemental Figure 1. MGMT and BMAL1 protein expression are rhythmic in human LN229 GBM cells *in vitro*, Related to Figure 1. A-D) Representative immunostaining of LN229 cells fixed every 4 hours for a period of 24 hours shows daily rhythms in MGMT (B) and BMAL1 (C) protein expression, both peaking at CT4, falling at CT8, reaching its trough at CT16, and rising at CT20 (n=6 frames per sample per time point). Scale bar is 20 μm.

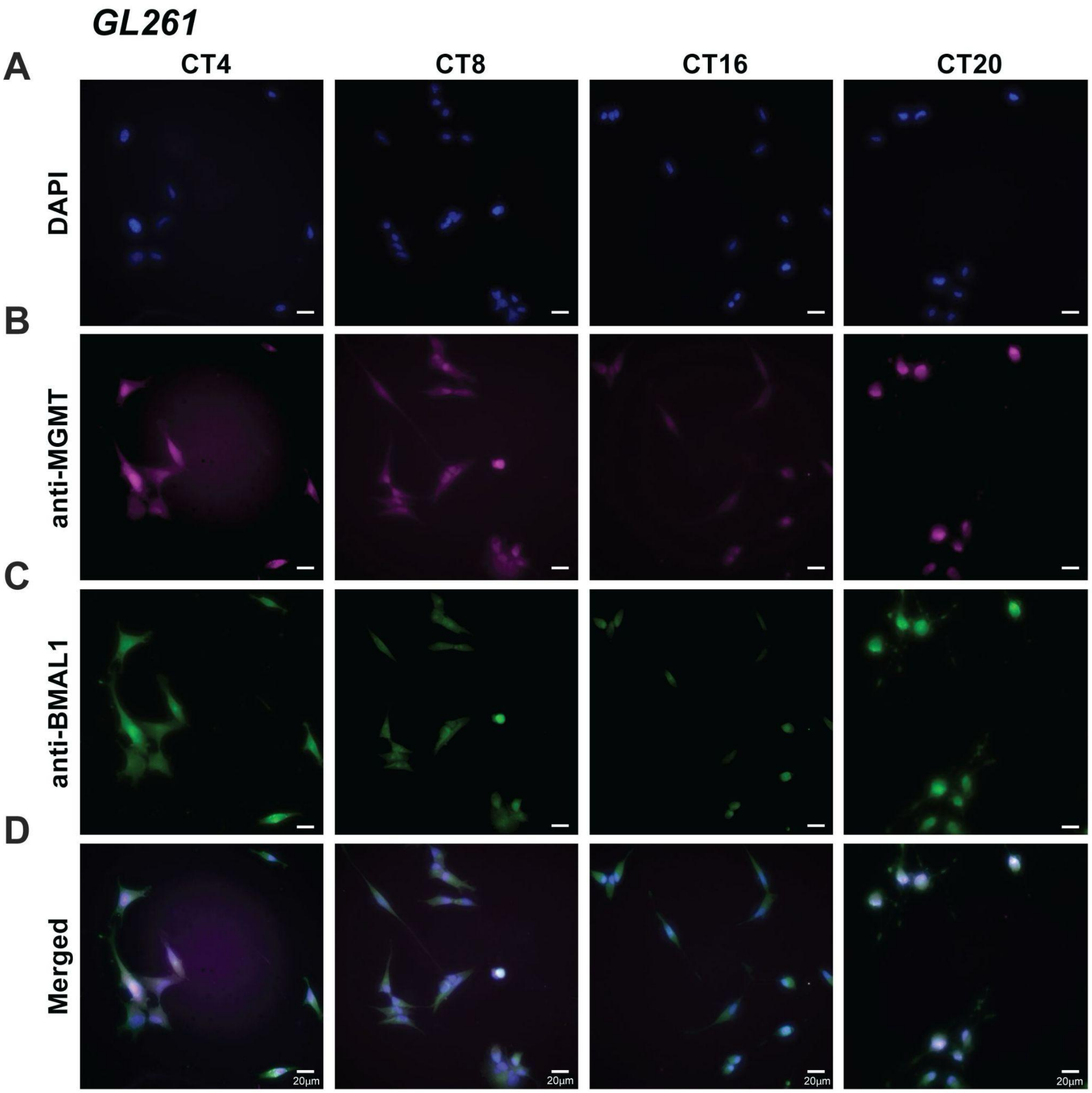

Supplemental Figure 2. MGMT and BMAL1 protein expression are rhythmic in murine GL261 GBM cells *in vitro*, Related to Figure 1. A-D) Representative immunostaining of GL261 cells fixed every 4 hours for a period of 24 hours shows daily rhythms in MGMT (B) and BMAL1 (C) protein expression, both peaking at CT4, falling at CT8, reaching its trough at CT16, and rising at CT20 (n=6 frames per sample per time point). Scale bar is 20 μm.

Details of the TMZ pharmacokinetics/pharmacodynamics

Two models were used to simulate the *in vitro* dynamics of TMZ arriving at the nucleus, one cell-based model and one simplified, to test robustness of the results. First, the cell-based model from (Corridore et al., 2025) was adapted for the dynamics of TMZ's conversion to MTIC. Parameter choices and model equations are provided below.

$$p_T = 0.003704$$

$$p_{T2} = 0.007785$$

$$k_{T0} = 1.1 \times 10^{-7}$$

$$\lambda_T = 2.086665$$

$$k_{M0} = 292$$

$$\lambda_M = 0.3107$$

$$\mathrm{pH}_0 = 7.2$$

$$V_{\mathrm{cell}} = 7 \times 10^{-12}\ \mathrm{L}$$

$$V_{\mathrm{in}} = 10^6 \cdot V_{\mathrm{cell}}$$

$$V_{\mathrm{out}} = 2 \times 10^{-3}\ \mathrm{L}$$

$$\mathrm{pH}_{\mathrm{extra}} = \mathrm{pH}_0$$

$$\mathrm{pH}_{\mathrm{intra}} = \mathrm{pH}_{\mathrm{extra}} - 0.01$$

$$k_{T,\mathrm{out}} = k_{T0} \cdot e^{\lambda_T \cdot \mathrm{pH}_{\mathrm{extra}}}$$

$$k_{M,\mathrm{out}} = k_{M0} \cdot e^{-\lambda_M \cdot \mathrm{pH}_{\mathrm{extra}}}$$

$$k_{T,\mathrm{in}} = k_{T0} \cdot e^{\lambda_T \cdot \mathrm{pH}_{\mathrm{intra}}}$$

$$k_{M,\mathrm{in}} = k_{M0} \cdot e^{-\lambda_M \cdot \mathrm{pH}_{\mathrm{intra}}}$$

$$\frac{d\,\mathrm{TMZ}_{\mathrm{out}}}{dt} = -\frac{p_T \cdot \mathrm{TMZ}_{\mathrm{out}} - p_{T2} \cdot \mathrm{TMZ}_{\mathrm{in}}}{V_{\mathrm{out}}} - k_{T,\mathrm{out}} \cdot \mathrm{TMZ}_{\mathrm{out}}$$

$$\frac{d\,\mathrm{TMZ}_{\mathrm{in}}}{dt} = \frac{p_T \cdot \mathrm{TMZ}_{\mathrm{out}} - p_{T2} \cdot \mathrm{TMZ}_{\mathrm{in}}}{V_{\mathrm{in}}} - k_{T,\mathrm{in}} \cdot \mathrm{TMZ}_{\mathrm{in}}$$

$$\frac{d\,\mathrm{MTIC}_{\mathrm{out}}}{dt} = k_{T,\mathrm{out}} \cdot \mathrm{TMZ}_{\mathrm{out}} - k_{M,\mathrm{out}} \cdot \mathrm{MTIC}_{\mathrm{out}}$$

$$\frac{d\,\mathrm{MTIC}_{\mathrm{in}}}{dt} = k_{T,\mathrm{in}} \cdot \mathrm{TMZ}_{\mathrm{in}} - k_{M,\mathrm{in}} \cdot \mathrm{MTIC}_{\mathrm{in}}$$

To demonstrate that the model results did not depend on the parameters in the in vitro TMZ PK/PD, a simple model was used in which MTIC in the nucleus was modeled as an impulse with a time delay:

$$\frac{dZ}{dt} = k_3 \delta(t - t_{\mathrm{Dose}} - t_{\mathrm{Delay}}) - k_4 Z$$

In this model, $\delta(t)$ captures a dosing strategy in which impulses of TMZ are given once at $t = t_{Dose}$ and reach the nucleus in toxin form, MTIC, after t_{Delay} . The half-life of TMZ is known to range from 70-90 minutes, establishing k_4, while k_3 can be chosen to be the dose amount through a rescaling of Z.

Predicting optimal timing with a simple mathematical model

Functionally, the cell-based model acts almost identically to the simple model (Figure S3), is able to reproduce the same global characteristics for both the thresholding effect, the dose-dependence of best time, and the parameter ranges that best match the time-free characteristics of prior data (Supplemental Figure 4 - 6).

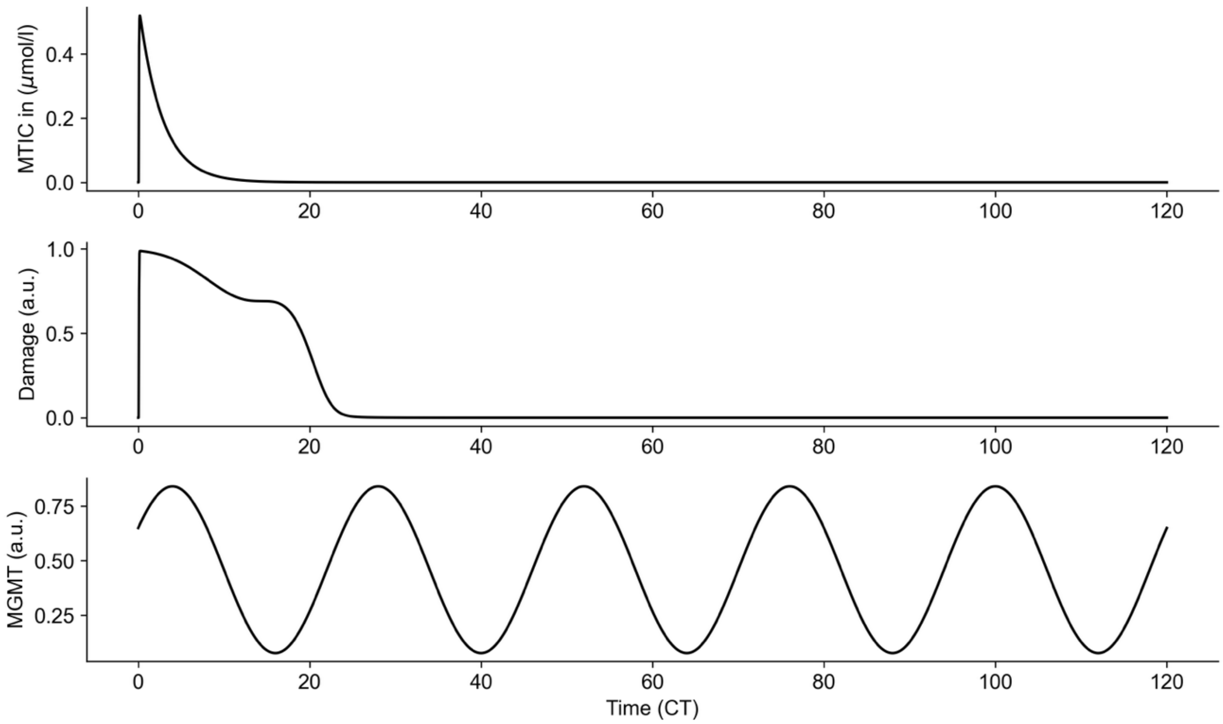

Supplemental Figure 3. The cell-based model closely resembles the simple model. Top row: MTIC in the nucleus, for an arbitrary pulse TMZ. Second row: DNA damage in the model. Third row: MGMT over time. The top row closely matches an impulse followed by exponential decay, as is the case in the simpler model.

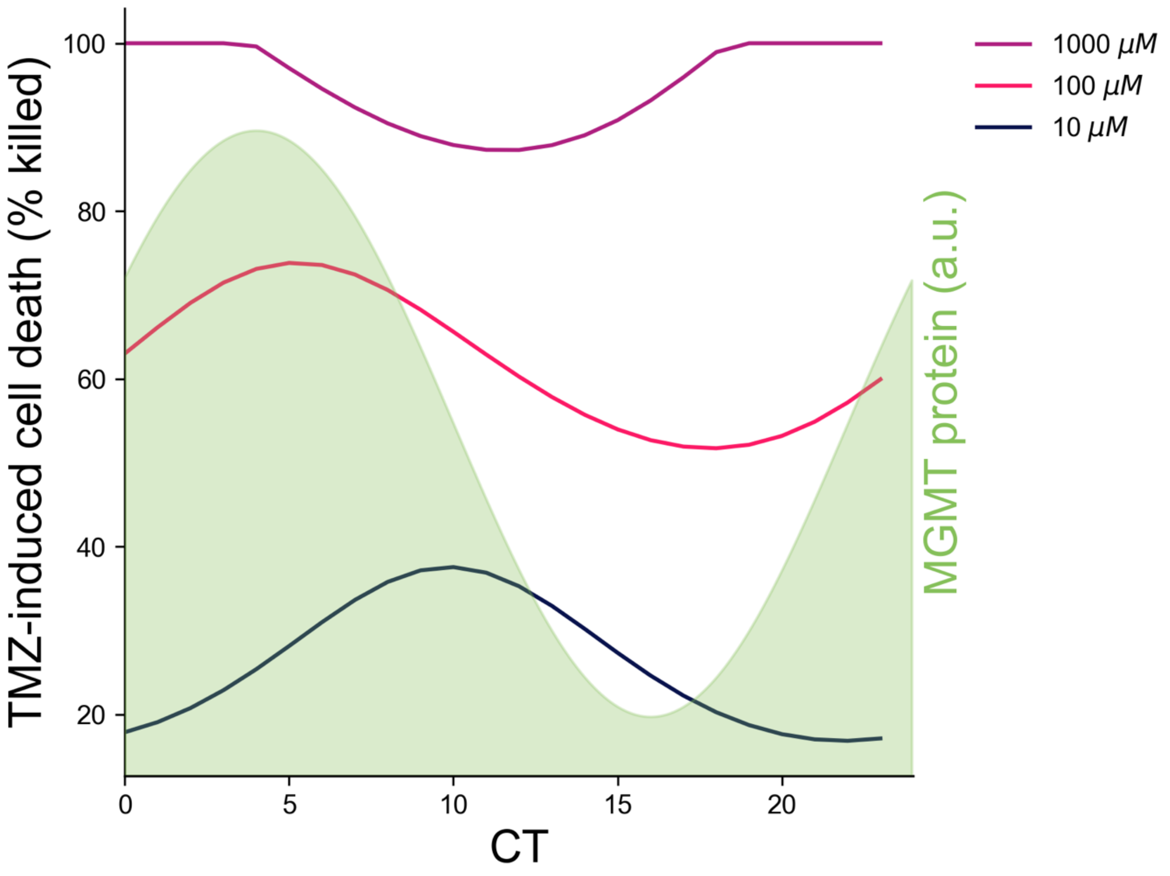

Supplemental Figure 4. An independent simple mathematical model validates maximum daily TMZ efficacy at the peak of MGMT expression, Related to Figure 2. Three different dose amounts and their time-of-day variation in the simpler model, consistently showing that efficacy of TMZ depends on dose and time since the daily peak in MGMT protein. Each line shows a computational model prediction of the fraction of GBM cell death *in vitro* following one of three doses of TMZ applied at different circadian times (CT). As previously found, the lowest concentration of TMZ (10 μM, black line) has a slightly smaller time-of-day rhythm in efficacy than the 100 μM dose (pink line), while the highest dose, 1000 μM (purple line), shows a reduced time-of-day effect due to a threshold past which 100% of cells are killed, masking greater rhythmicity. The best time for the 100 μM dose in this theoretical simulation is CT5. The

previously observed rhythm in MGMT protein abundance is superimposed as an indicator of time of day and daily MGMT expression (green shaded background).

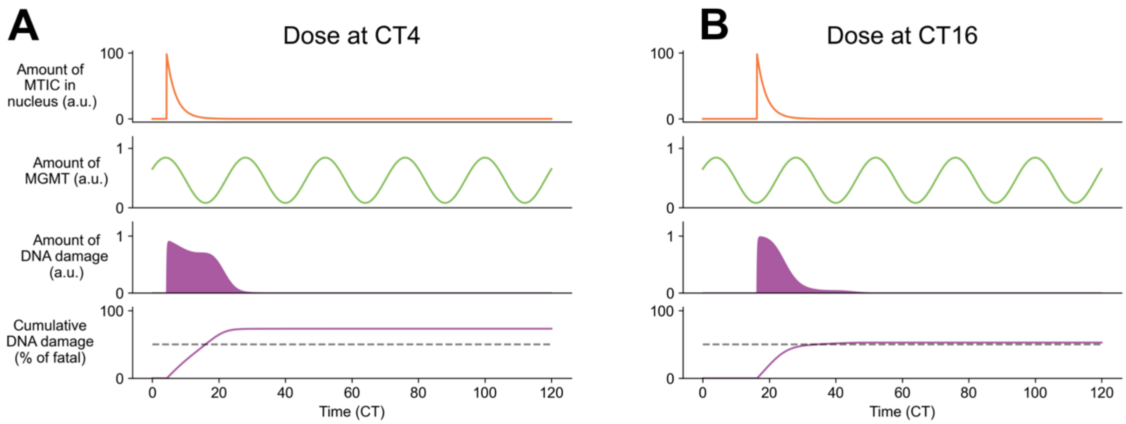

Supplemental Figure 5. Simple mathematical model of dosing with TMZ at either maximum (CT4) or lowest (CT16) MGMT protein abundance predicts superior results at CT4, Related to Figure 3. A) Simple mathematical modeling that incorporates daily rhythms in TMZ delivery (first row) and MGMT protein abundance (second row) finds increased DNA damage (third row) and cumulative cell death (fourth row) when TMZ is delivered at CT4, corresponding to the peak or falling phase of MGMT expression. The amount of DNA damage is predicted to persist for a longer time and pass a 50% threshold, leading to greater TMZ-induced GBM cell death. B) Dosing at CT16, time of lowest MGMT protein abundance, yields a decreased amount of predicted DNA damage and cell death. The amount of predicted DNA damage lasts for a shorter time period and barely crosses a 50% threshold, leading to reduced TMZ-induced GBM cell death. The top row in each plot shows the time profile of the toxin form of TMZ (MTIC) in the nucleus, the second row shows MGMT protein, the third shows the damage to the DNA, and the fourth shows cumulative damage to the DNA, scaled by the fatal threshold D_T. The area under the curve (purple, third row) changes with changing dose times. While the simple model describes cumulative damage at the level of a single cell, we can

equivalently think of this damage as the probability of cell death in a population of tumor cells, with higher cumulative damage meaning greater probability of cells in the population dying.

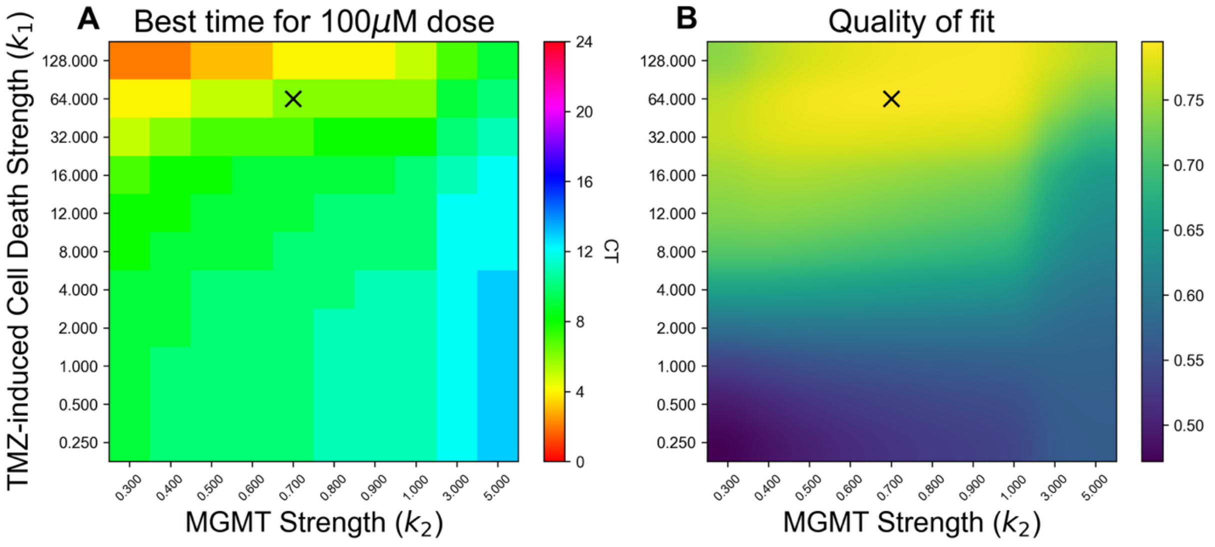

Supplemental Figure 6. Maximum TMZ efficacy and cell death is obtained with morning treatment for parameters that best match the experimental data as predicted by a simple mathematical model, Related to Figure 4. A) Using a simple modeling that incorporates the strength of TMZ-induced DNA damage (y-axis) relative to the daily ability of MGMT to repair DNA (x-axis) shows that, across all the parameters tested that correspond to high quality fits (yellow region in B), the dose time that maximizes cell damage is near the peak of MGMT (CT4) or on its descent, corresponding to morning dosing. No optimal TMZ dosing times were found near the lowest of MGMT expression (dark blue, purple in the color bar) among the parameter sets tested. B) Heatmap showing which parameter choices best match the average DNA damage for dose quantities and the extent of dosing time variation in previously reported data. Yellow indicates a closer fit to the data, while blue indicates a worse fit. The X marks in A and B correspond to the parameter set that best matches the mean cell death and range of cell death observed in experimental data**.**

Addition of a Michaelis-Menten term

To further explore model dynamics, Michaelis-Menten term was added to the DNA damage equation. Supplemental Figures 7-9 show sample outputs for the cell-based model with a Michaelis-Menten ($\alpha = 0.7$) term, as specified below:

$$\frac{dD}{dt} = k_1 \mathrm{MTIC_{in}}(1 - D) - \frac{k_2 M D}{a + D}$$

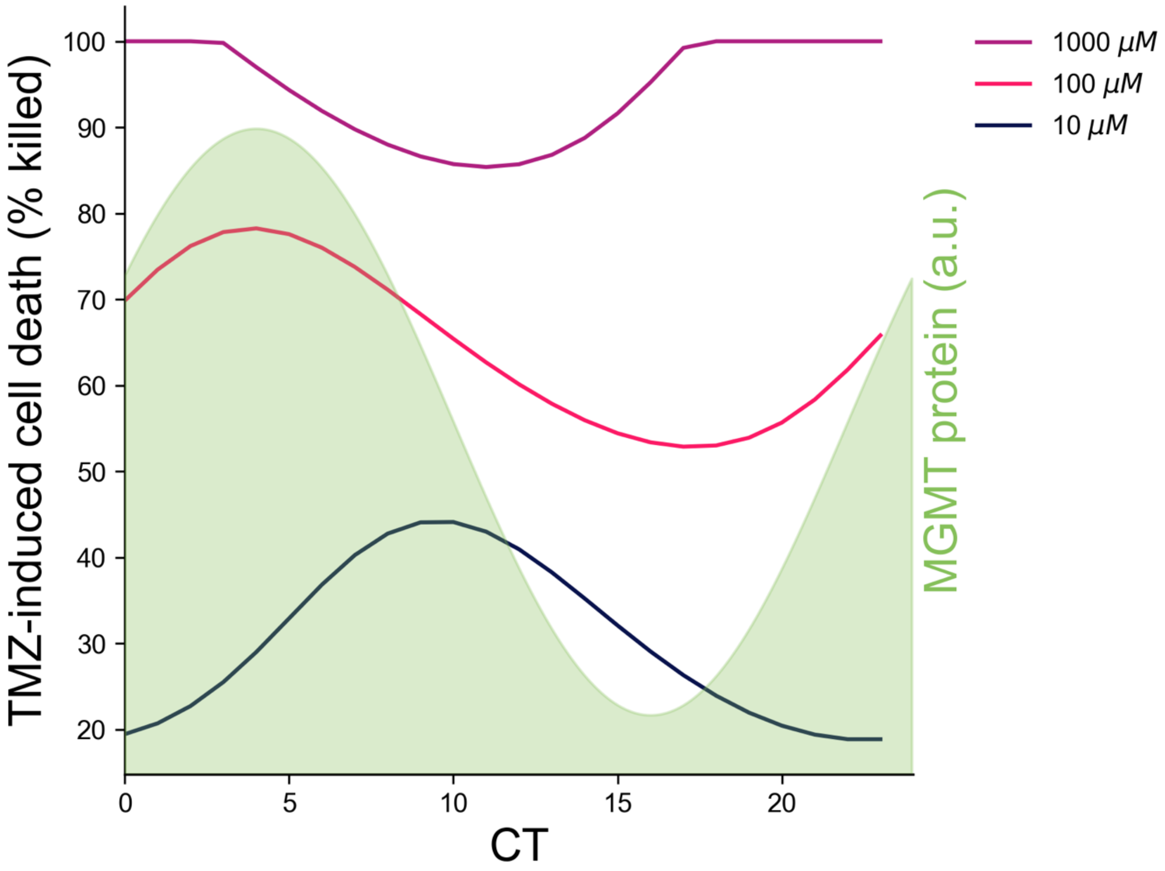

Supplemental Figure 7. Three different TMZ dose amounts and their time-of-day variation in the cell-based model equipped with a Michaelis-Menten term, consistently show that TMZ efficacy depends on dose and time since the daily peak in MGMT protein abundance, Related to Figure 2. Each line shows a computational model prediction of the fraction of GBM cell death *in vitro* following one of three doses of TMZ applied at different

circadian times (CT). As previously found, the lowest concentration of TMZ (10 μM, black line) has a slightly smaller time-of-day rhythm in efficacy than the 100 μM dose (pink line), while the highest dose, 1000 μM (purple line), shows a reduced time-of-day effect due to a threshold past which 100% of cells are killed, masking greater rhythmicity. The best time for the 100 μM dose in this theoretical simulation is CT4. The previously observed rhythm in MGMT protein abundance is superimposed as an indicator of time of day and daily MGMT expression (green shaded background).

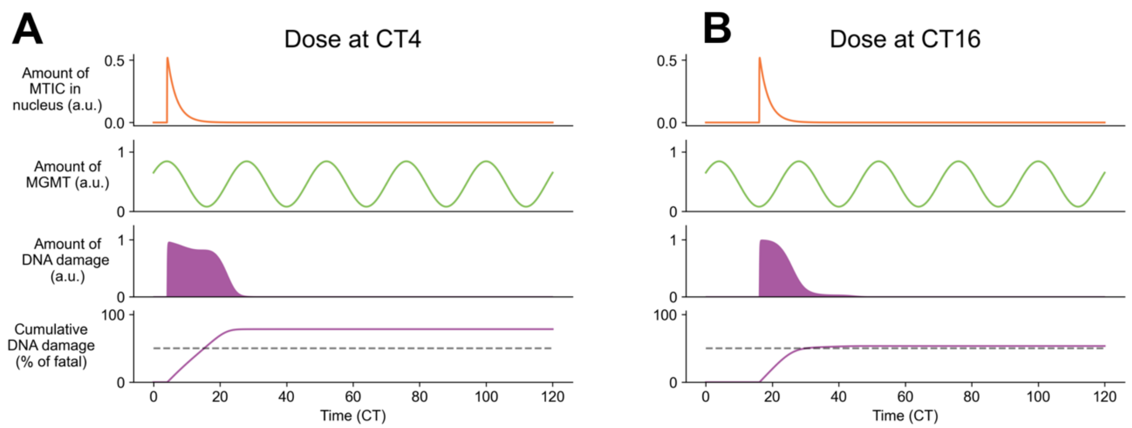

Supplemental Figure 8. Incorporating a Michaelis-Menten term into the cell-based model yields findings that dosing at CT4 is superior to dosing at CT16, Related to Figure 3. A) Simple mathematical modeling that incorporates daily rhythms in TMZ delivery (first row) and MGMT protein abundance (second row) finds increased DNA damage (third row) and cumulative cell death (fourth row) when TMZ is delivered at CT4, corresponding to the peak or falling phase of MGMT expression. The amount of DNA damage is predicted to persist for a longer time and pass a 50% threshold, leading to greater TMZ-induced GBM cell death. B) Dosing at CT16, time of lowest MGMT protein abundance, yields a decreased amount of predicted DNA damage and cell death. The amount of predicted DNA damage lasts for a shorter time period and barely crosses the 50% threshold, leading to reduced TMZ-induced

GBM cell death. The top row in each plot shows the time profile of the toxin form of TMZ (MTIC) in the nucleus, the second row shows MGMT protein, the third shows the damage to the DNA, and the fourth shows cumulative damage to the DNA, scaled by the fatal threshold D_T. The area under the curve (purple, third row) changes with changing dose times. While the simple model describes cumulative damage at the level of a single cell, we can equivalently think of this damage as the probability of cell death in a population of tumor cells, with higher cumulative damage meaning greater probability of cells in the population dying.

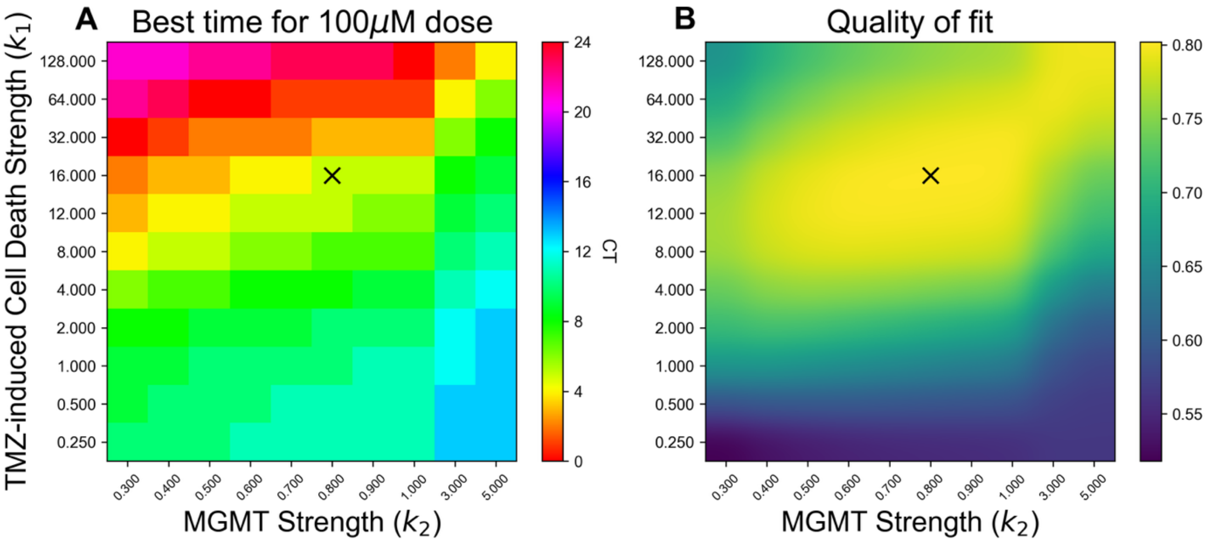

Supplemental Figure 9. Maximum TMZ efficacy and cell death is obtained with morning treatment for parameters that best match the experimental data in cell-based model equipped with a Michaelis-Menten term for DNA damage, Related to Figure 4. A) Using a model equipped with a Michaelis-Menten term that incorporates the strength of TMZ-induced DNA damage (y-axis) relative to the daily ability of MGMT to repair DNA (x-axis) shows that, across all the parameters that correspond to high quality fits (yellow region in B), the dose time that maximizes cell damage is near the peak of MGMT (CT4) or on its descent, corresponding to morning dosing. No optimal TMZ dosing times were found near the lowest of MGMT expression (dark blue, purple in the color bar). Several parameter sets (pink, upper left corner)

corresponded to optimal dosing as MGMT rises to its maximum (CT20), but these parameters were poor fits to the data, unable to match the mean and range of previously reported cell death percentages as a function of dosing time. B) Heatmap showing which parameter choices best match the average DNA damage for dose quantities and the extent of dosing time variation in previously reported data. Yellow indicates a closer fit to the data, while blue indicates a worse fit. The X marks in A and B correspond to the parameter set that best matches the mean cell death and range of cell death observed in experimental data.

A different waveform for MGMT

To pressure test the assumption of MGMT being a sinusoid, an alternative waveform was tested, with a spikier MGMT peak resembling the left plot of Figure 1F. Full details of the alternative waveform can be found on the GitHub repository accompanying this paper.

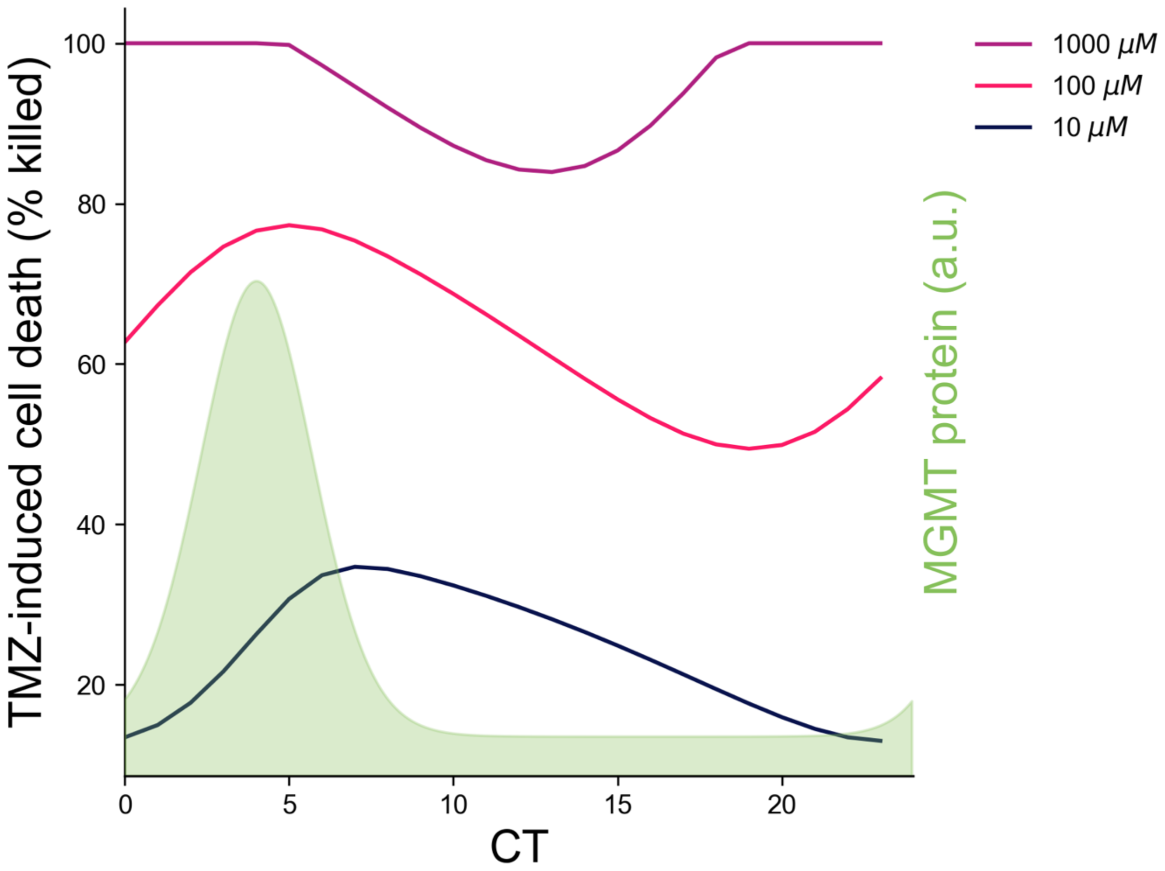

Supplemental Figure 10. Three different TMZ dose amounts and their time-of-day variation in the cell-based model equipped with a non-sinusoidal waveform for MGMT consistently show that TMZ efficacy depends on dose and time since the daily peak in MGMT protein abundance, Related to Figure 2. Each line shows a computational model prediction of the fraction of GBM cell death *in vitro* following one of three doses of TMZ applied at different circadian times (CT). As previously found, the lowest concentration of TMZ (10 μM, black line) has a smaller time-of-day rhythm in efficacy than the 100 μM dose (pink line), while the highest dose, 1000 μM (purple line), shows a reduced time-of-day effect due to a threshold past which 100% of cells are killed, masking greater rhythmicity. The best time for the 100 μM dose in this theoretical simulation is CT5. The "spikier", non-sinusoidal rhythm in MGMT protein

abundance is superimposed as an indicator of time of day and daily MGMT expression (green shaded background).

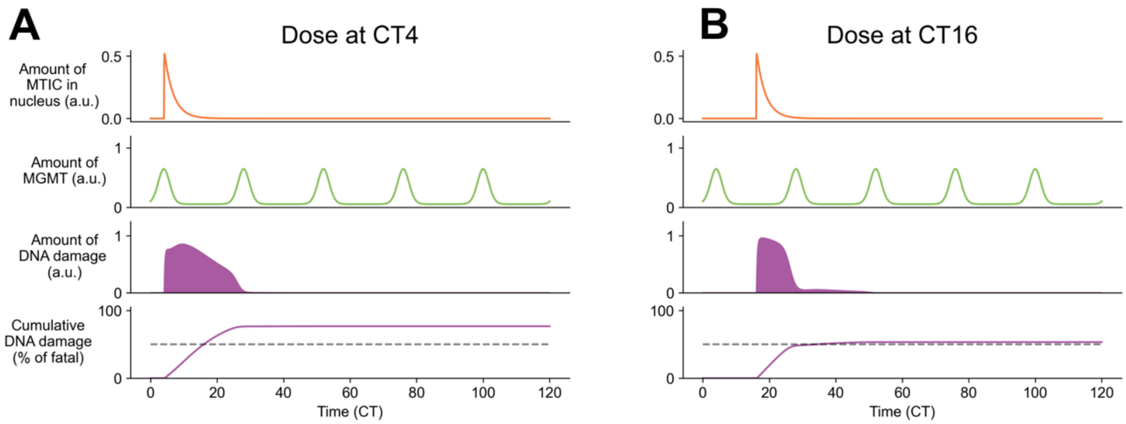

Supplemental Figure 11. Replacing the sinusoidal MGMT with a spikier waveform in the cell-based model yields findings that dosing at CT4 is superior to dosing at CT16, Related to Figure 3. A) Simple mathematical modeling that incorporates daily rhythms in TMZ delivery (first row) and a spikier waveform for MGMT protein abundance (second row) finds increased DNA damage (third row) and cumulative cell death (fourth row) when TMZ is delivered at CT4, corresponding to the peak or falling phase of MGMT expression. The amount of DNA damage is predicted to persist for a longer time and pass a 50% threshold, leading to greater TMZ-induced GBM cell death. B) Dosing at CT16, time of lowest MGMT protein abundance, yields a decreased amount of predicted DNA damage and cell death. The amount of predicted DNA damage lasts for a shorter time period and barely crosses the 50% threshold, leading to reduced TMZ-induced GBM cell death. The top row in each plot shows the time profile of the toxin form of TMZ (MTIC) in the nucleus, the second row shows (non-sinusoidal) MGMT protein, the third shows the damage to the DNA, and the fourth shows cumulative

damage to the DNA, scaled by the fatal threshold D_T. The area under the curve (purple, third row) changes with changing dose times. While the simple model describes cumulative damage at the level of a single cell, we can equivalently think of this damage as the probability of cell death in a population of tumor cells, with higher cumulative damage meaning greater probability of cells in the population dying.

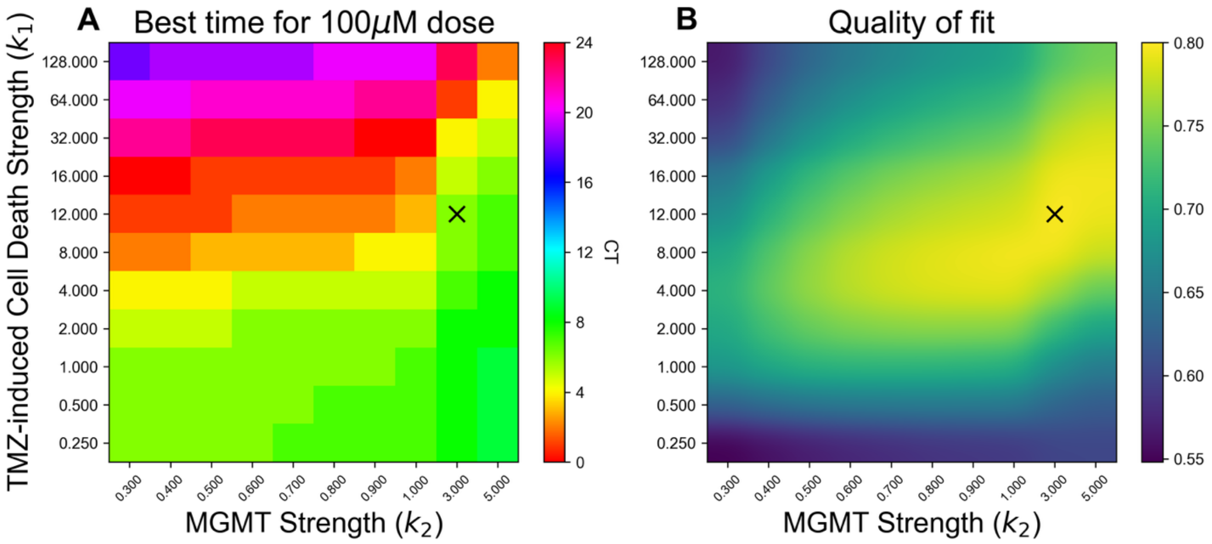

Supplemental Figure 12. Maximum TMZ efficacy and cell death is obtained with morning treatment for parameters that best match the experimental data in a cell-based model equipped with a spikier waveform assumption for MGMT dynamics, Related to Figure 4. A) Using a model equipped with a spikier (non-sinusoidal) MGMT waveform that incorporates the strength of TMZ-induced DNA damage (y-axis) relative to the daily ability of MGMT to repair DNA (x-axis) shows that, across all the parameters that correspond to high quality fits (yellow region in B), the dose time that maximizes cell damage is near the peak of MGMT (CT4) or on its descent, corresponding to morning dosing. While several parameter sets (upper left corner) yielded optimal TMZ dosing times near the lowest part of MGMT expression (dark blue, purple in the color bar), none were good fits for the data. B) Heatmap showing which parameter choices best match the average DNA damage for dose quantities and the extent of dosing time

variation in previously reported data. Yellow indicates a closer fit to the data, while blue indicates a worse fit. The X marks in A and B correspond to the parameter set that best matches the mean cell death and range of cell death observed in experimental data.
